# Systematic reviews and the *Journal of Antimicrobial Chemotherapy;* past, present and future. A systematic reappraisal

**DOI:** 10.1093/jac/dkaf282

**Published:** 2025-08-21

**Authors:** James C Hurley

**Affiliations:** Melbourne Medical School, University of Melbourne, Parkville, Victoria, Australia; Ballarat Clinical School, Deakin University, Ballarat, Victoria, Australia; Internal Medicine Service, Ballarat Health, Grampians Health Services, Ballarat, Victoria, Australia

## Abstract

Since 2003, >200 systematic reviews (SRs) have been published in the *Journal of Antimicrobial Chemotherapy* (*JAC*). Many have been widely cited. Guidelines for proper execution were outlined in 2005. Since then, new threats, challenges and methods have emerged. Data provenance is an emerging threat. There are several assumptions and limitations of both SR methods and the primary studies they include. The pivotal impact of these assumptions is illustrated using 13 SRs of pneumonia prevention in ICU patients on inferences towards the aspirational goal of ‘Pneumonia zero’ as a case study. Depending on these assumptions, the SRs of antimicrobial-based interventions pivot between two not incompatible inferences of pneumonia prevention for individual ICU patients versus harm for ICU populations. The case study highlights how newer methods for data visualization enhance reader insight into the underlying data beyond the summary effect size. To remain relevant to evidence-based medicine, SRs will need to adapt to emerging challenges and to recognize and validate the key underlying assumptions. This is especially so for antimicrobial-based infection prevention interventions given their potential for spillover effects.

## Introduction

There have been >200 systematic reviews (SRs) published in the *Journal of Antimicrobial Chemotherapy* (*JAC*) over the past 20 years. A SR attempts to identify, appraise and synthesize all the empirical evidence that meets pre-specified eligibility criteria to answer specific research questions. Researchers conducting SRs use explicit, systematic methods to minimize bias, in producing robust findings to inform decision making.^1^Statistical methods (meta-analysis) may or may not be used to analyse and summarize the results of the included studies. SRs are placed high in the evidence-based hierarchy.

When this topic was previously reviewed in 2005, fewer than 10 SRs had appeared in *JAC* and both ‘systematic review’ and ‘meta-analysis’ were novel techniques applied in deriving summary estimates of treatment effects from published randomized controlled trials (RCTs) towards the development of evidence-based treatment guidelines.^2^ Today there would be few interventions examined in an RCT that have not been included within several SRs.

The expectations for how SRs should be properly undertaken has been considered previously. Since then, new challenges have emerged that have led some to question the value of SRs and how they might best serve evidence-based medicine.^3–5^ Some have stated that ‘…a sizeable proportion are flawed beyond repair…’^3^ Checklists, such as the PRISMA2020 checklist, improve the reproducibility of an SR.^6^

This review identifies several emerging challenges and suggests that better methods of displaying and contextualizing the study results with external benchmarks will be required for SRs to remain high within the evidence hierarchy.

## Past

**Figure 1.**
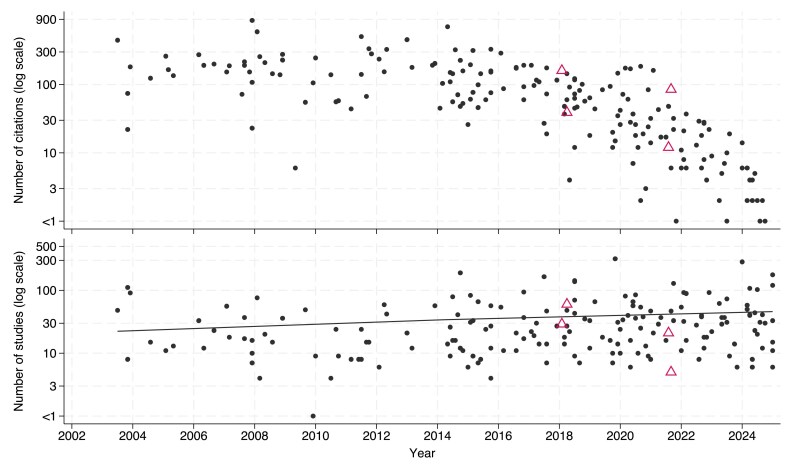
SRs (shown with black circles) and network meta-analyses (shown with triangles) published in the *JAC* between 2003 and 2024 with the number of eligible studies that each included (bottom; together with trend line derived by unweighted linear regression) and the number of citations each received (top). Note the *y*-axis is logarithmic, truncated at 1.

Here, I review some SRs published in *JAC*. The search, and citation count, were obtained using Google scholar for publications in *JAC* with the term ‘systematic review’ in the title (Figure 1). Half of the SRs have appeared in only the past 7 years with 11 relating to COVID.

Several were highly cited with 17 generating follow up comment or correspondence in the journal. This suggests that SRs addressing interventions that are either controversial, emerging or for which the evidence is mixed are of great interest.

One of the earliest SRs examined the evidence for the association of specific antibiotics with hospital-acquired *Clostridium difficile*-associated diarrhoea. This SR (cited >400 times) noted that many of the primary studies had limitations with use of incorrect control groups, the presence of bias, inadequate control of confounding and small sample sizes and hence was unable to establish causal relationships.^7^

The most widely cited SR (>850 citations) examined the association of extended-spectrum β-lactamase production on mortality and the delay in effective therapy in Enterobacteriaceae bacteraemia. This SR likewise acknowledged that the lack of controlled studies limits any interpretation regarding causality in this association.^8^

The SR with the most included studies (*n* = 318) examined the question of drug–drug interactions among patients receiving antibacterials for COPD exacerbations. As the authors note, many of the included studies were observational in nature.^9^

One notable SR examined the question of drug dosing for septic patients with acute renal failure receiving continuous renal replacement therapy. This SR found that out of 64 potentially eligible studies, none reported sufficient pharmacokinetic information, such as volume of distribution and clearance, on which to base drug dosing regimens.^10^ The paucity of suitable RCTs on which to base clinical practice guidelines has been noted elsewhere.^11^

Two SRs examined diagnostic approaches for the rapid detection of antimicrobial resistance in *Mycobacterium tuberculosis*.^12,13^ These meta-analyses used summary receiver operating characteristic curves to summarize the diagnostic accuracy.^14^

Other SRs address questions relating to aetiology, prevalence and adverse effects as well antimicrobial chemotherapy used as prevention or treatment of infectious diseases for individual patients.

Several SRs have addressed research questions related to populations. For example, a SR including >170 studies of carbapenem resistance among clinical isolates of *Acinetobacter baumannii* was able to display the estimated resistance rates by country on a map of the world.^15^

## Present

The poor quality of the primary studies, as highlighted by several examples before, limits the validity of SRs. Traditionally, SRs have address research questions framed using PICO components to specify the population (P), intervention (I), comparison (C) and outcome (O). The ideal study design towards estimating causal effects of interventions is an RCT, adequately powered and free from bias. The few published studies attaining this ideal are generally outnumbered by those that are either non-randomized, uncontrolled or studies with before–after designs. What should an SR do with these non-RCT observations?

A serious challenge, which goes beyond SRs, is fraudulent data. There is no simple solution to this problem except constant vigilance by authors, editors, reviewers and readers.^16–18^

Another challenge is the increasing number of potential studies that an SR might include. There have been 12 SRs in *JAC* with >100 included studies of which 11 were published in the last decade. How best to present large amounts of data has emerging solutions. For example, an SR of antibiotic prescribing in COVID-19 patients in China including >250 studies used violin plots, which are a hybrid of a box plot and a kernel density plot, to summarize the large amount of study data to enable visual contrasts of the distribution of numeric data across several groups.^19^

Even within studies that achieve the RCT ideal, there may be either more than one treatment group or more than one control group. Often SRs will drop these additional arms from the analysis. Is this loss of information, sometimes substantial, avoidable?

Which approach to meta-analysis is best? A viewpoint regarding the safety of cefipime use as part of empiric therapy for febrile neutropenia highlighted that reanalysing data published by the US FDA using Bayesian methods of meta-analysis leads to inferences contrary to that obtained using the traditional frequentist methods.^20^ This potential for differing inferences from Bayesian versus frequentist methods applied to the same data is not unique to meta-analysis.^21^ Frequentist methods derive inference from imaginary repetitions of the same experiment projected as a data distribution. With Bayesian methods, by contrast, the inference is derived from the investigator’s prior belief, whether enthusiastic versus sceptical, as modified by the observed data.^21^

There are many important research questions that would be difficult to address with an RCT, such as those relating to potential adverse effects or resulting in patient harm, as in the cefipime example, or where the outcome in question is rare. How do we assess evidence towards the development of guidelines for these questions?

## Stable unit treatment variable assumption

Research questions estimating intervention effects for individuals use an RCT design wherein individual patients are randomized. Estimating intervention effects for populations requires a cluster randomized trial (CRT) design wherein whole populations are randomized.^22^ The RCT versus CRT approach will answer the same research question at different levels, a distinction that is especially relevant for research questions relating to the population effects of antimicrobial chemotherapy.^23^ For example, the potential for acquiring resistance in the population deserves more attention in SRs of antimicrobial use.^24^

Of note, the CRT, but not the RCT, avoids any spillover of intervention effect between control and intervention groups. Any potential for spillover invalidates the stable unit treatment variable assumption (SUTVA). SUTVA, although fundamental to causal inference conferred by randomized treatment allocation, is rarely mentioned.

SUTVA can only be verified by referencing data external to the study. For example, RCTs of vaccine interventions for transmissible infections might show seemingly ineffective effect sizes (ESs), as spillover of prevention effect to concurrent unvaccinated control groups bias the study result towards the null. Observing lower infection rates in both control and intervention groups versus comparable non-exposed populations suggests spillover of prevention effects occurred within the RCT.^25^ Conversely, the potential for spillover of harmful effects from intervention to control groups should also be considered, as this could inflate the apparent ES away from the null.

## Risk of bias

The summary findings within SRs are increasingly accompanied by certainty ratings based on assessments of risk of bias (ROB), including the directness of the evidence, the consistency of the results, the precision of the estimates and the risk of publication bias. How reliable are these certainty ratings in addressing the inclusion of suboptimal studies within SRs? Moreover, certainty is a loaded term that implies certainty in the findings beyond merely observations deemed to be reliable.

The ROB assessed for each individual study includes elements such as sequence generation and allocation concealment (selection bias), blinding of participants and providers (performance bias), blinding of outcome assessors (detection bias), incomplete outcome data (attrition bias) and selective outcome reporting (reporting bias). The process of assessing ROB for both randomized and non-randomized studies is comprehensively detailed in *The Cochrane Handbook*.^26^ Of note, the ROB does not assess SUTVA, as this requires reference to observations external to the RCT.

ROS scores may provide a basis for exploring how the study ES estimates vary by study quality. This may be especially relevant for study end points that are subjective, such as the diagnosis of ventilator associated pneumonia (VAP) among mechanically ventilated ICU patients.^27^

## Heterogeneity and *I*^2^

Heterogeneity within SRs is often represented by the *I*^2^ metric, which is the proportion of total variability in ES due to between study heterogeneity and judged on a scale with arbitrary levels of concern. The *I*^2^ metric is widely misunderstood.^28,29^  *I*^2^ is not an absolute measure of heterogeneity. Being a proportion, *I*^2^ is a relative measure. Heterogeneity is poorly estimated where there are few studies and provides false reassurance when heterogeneity appears absent. The L’abbe plots display the relationship between event rates in the control and intervention groups and provide a visual indication of heterogeneity. However, as with the *I*^2^ metric, heterogeneity inferences for the same data in a L’abbe plot will differ depending on whether the ES is derived as odds ratios, risk ratios or risk differences.^30^

A better guide than the *I*^2^ metric to gauge heterogeneity among the individual study results within a meta-analysis is the 95% prediction interval (95% PI) associated with the summary ES.^31,32^ The 95% PI incorporates the between study variance (tau^2^) in its calculation and is wider than the 95% confidence interval (95% CI). The 95% PI indicates where the ES of 95 of the next hypothetical 100 studies would probably be found whereas the 95% CI, which is narrower than the 95% PI, represents the limits between which 95 of the next hypothetical 100 replications of summary ES estimates would be expected to lie.

## Contrast- versus arm-based meta-analysis

Traditionally, the meta-analysis ES of interest arise from the contrast between the pair of singleton control and intervention groups within each RCT. For data originating from ‘ideal’ RCT designs, a causal inference can be inferred from this contrast-based analysis. Increasingly there is a need to include studies that may have multiple treatment or control groups. Techniques are emerging to accommodate these multi-arm studies in arm-based analyses. One recent example is a network meta-analysis (NMA) of data from 70 multi-arm studies that examined whether vancomycin plus piperacillin/tazobactam increases nephrotoxicity compared with other anti-pseudomonal beta-lactams such as cefipime or meropenem.^33^

The merits and limitations of contrast-based versus the emerging arm-based methods are controversial but the latter are gaining increasing acceptance.^34^ The strength of contrast-based methods relies on the causal inference conferred by random assignment, which is lost in an arms-based analysis. Of note, arm-based methods are accepted and widely used in the meta-analysis of diagnostic tests as the interest is usually in the arm-based properties of a test, being summary sensitivity and specificity.^14^

## A case study: ‘VAP zero’

The findings of several SRs of >200 RCTs suggest that VAP is preventable among mechanical ventilated ICU patients through various intervention methods.^35–47^ The summary findings of 13 Cochrane reviews of VAP prevention interventions imply that topical antibiotic prophylaxis (TAP), with protocolized parenteral antibiotic prophylaxis (PPAP) as selective digestive decontamination (SDD) or without PPAP, as selective oropharyngeal decontamination, is superior to topical chlorhexidine which in turn is superior to various non-antimicrobial interventions, which are summarized here as a composite (Table 1). Moreover, the TAP + PPAP combination appears to prevent mortality, a finding that is rated with high certainty. Are inferences from these antimicrobial SRs safe? Moreover, do they allow projections towards attaining ‘zero VAP’ among ICU populations?^49^

**Table 1.** Summary results from 13 SRs of interventions to prevent pneumonia and mortality in ICU patients

SR^a^	Patients	Studies	Median event rate (per 1000 patients)		Effect size				ITT/PP
	(*n*)	(*n*)	Control	Intervention	RR	95% CI	*I* ^2^ (%)	Certainty	
Pneumonia prevention									
Non-antimicrobial									
Composite^35–43^	24 594	122	75–314	48–268	0.82	0.71–0.93	50	Low to moderate	ITT
Chlorhexidine									
Hua^44^	2451	18	243	180	0.75	0.62–0.91	35	High	ITT
Zhao^45^	1206	13^b^	261	175	0.67	0.47–0.97	66	Moderate	ITT
TAP + PPAP^c^									
Liberati^46^	3024	16	NR	NR	0.28	0.2–0.38	56	NS^d^	ITT
Minozzi^47^	2951	17	417	179	0.43	0.35–0.53	55	Moderate	PP
TAP alone^c^									
Liberati^46^	1735	12	NR	NR	0.34	0.21–0.55	70	NS^d^	ITT
Minozzi^47^	1848	13	324	162	0.5	0.36–0.69	66	Low	PP
Mortality prevention									
Chlorhexidine									
Hua^44^	2014	14	222	242	1.09	0.96–1.23	0	Moderate	ITT
Zhao^45^	944	9^b^	190	247	1.03	0.8–1.33	0	Moderate	ITT
TAP + PPAP^c^									
Liberati^46^	4075	17	NR	NR	0.75	0.65–0.87	0	NS^d^	ITT
Minozzi^47^	5290	18	303	225	0.84	0.73–0.96	40	High	PP
TAP alone^c^									
Liberati^46^	1783	13	NR	NR	0.97	0.79–1.2	0	NS^d^	ITT
Minozzi^47^	3274	15	305	296	0.97	0.87–1.07	0	Moderate	PP

^a^Nine SRs of pneumonia prevention in ICU patients using gastrointestinal,^35^ feeding,^36–38^ airway^39–42^ and probiotic^43^ based interventions are presented here as a composite. None of these interventions achieved significant mortality prevention. The median event rate is a range among the nine SRs and the composite ES estimate is from.^48^

^b^Eight studies included in Hua meta-analysis were excluded from corresponding meta-analysis in Zhao^45^ (see Table S1).

^c^Results in many TAP studies were published as per protocol (PP) and used by Minozzi^47^ whereas the intention to treat (ITT) data were obtained as personal communications by Liberati from study authors (Tables S2 and S3).

^d^For Liberati,^46^ certainty is not stated (NS) except that publication bias was considered unlikely on the basis of the appearance of the funnel plot for the VAP prevention ES distribution.

The methods, techniques of literature searching, ROB assessments and data extraction among these SRs, being as prescribed by *The Cochrane Handbook*,^1^ are exemplary. Furthermore, Cochrane SRs are distinguished by their rigorous attention to detail, providing study data that allows for the replication of each meta-analysis in both contrast-based and arms-based analyses.^48,50–53^

Among Cochrane reviews relating to VAP prevention are two pairs relating to topical chlorhexidine, appearing in 2017 and 2020, and two relating to TAP ± PPAP, appearing in 2009 and 2021. Each pair included similar primary studies and share authorship. There are two specific minor concerns and five major concerns (Tables S1–S3, available as Supplementary data at *JAC* Online). Moreover, several underlying assumptions differ, which illustrate how inferences from these SRs pivot on specific, and unstated, assumptions.

First, of minor concern, the more recent SR of topical chlorhexidine excludes or reclassifies eight studies included in the earlier SR without ROB concerns identified. While these exclusions are acknowledged in the more recent SR, the resulting summary VAP prevention ES calculated is slightly magnified, but the certainty is downrated.

**Figure 2.**
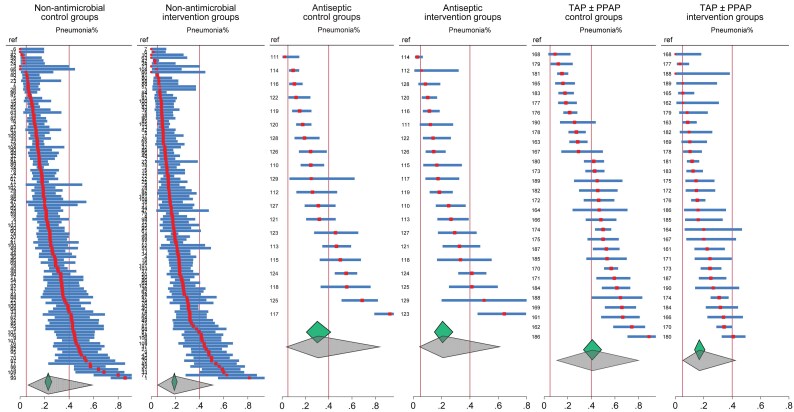
Caterpillar plots of VAP incidence proportions (and associated study specific 95% confidence intervals) from control and intervention arms of RCTs as abstracted within 13 Cochrane reviews of non-antimicrobial,^35–43^ antiseptic^44,45^ and topical antibiotic (TAP with or without PPAP)^46,47^ based methods of VAP prevention in ICU patients. The vertical lines represent the upper (right) and lower (left) limits of VAP incidence from expert opinion.^54–56^ The summary proportion and associated 95% confidence interval (small diamonds) and 95% prediction interval (larger diamonds) as derived by random effects meta-analysis are shown. Note that, being caterpillar plots, the study arms are arranged in order of the VAP incidence proportion, and the order of studies differs between control and intervention arms. The study numbers are as listed in.^48^ The respective *I*^2^ for the control and intervention groups are 86.5 and 82.8, 91.7 and 84.8, and 92.4 and 81.6 for the non-antimicrobial, antiseptic, and topical antibiotic studies, respectively. Duplex studies, where control group patients received an antimicrobial intervention, have been excluded.

Second, the heterogeneity metrics associated with all summary ESs substantially misrepresent the study heterogeneity. These metrics, which appear acceptable, merely estimate the statistical heterogeneity in each ES. With studies of >15 regimens among diverse patient populations and ICU settings included among these SRs, which are the summary ES representative of? Moreover, the substantial dispersion in VAP proportion among the component groups has not been considered (Figure 2).

The first major concern is that the most recent SR of TAP + PAPP includes one large CRT^57^ of TAP + PPAP (risk ratio ES 1.01; 0.87–1.16), which contributes 14% by weight to, but differs significantly from, the overall mortality prevention summary ES (0.84; 0.73–0.96), which is rated with high certainty. This discrepancy is not discussed within this SR. Is this high certainty rating justified if the summary ES fails to predict the results of the largest component study?

Likewise, another SR^58^ found the summary ES for mortality prevention derived from 27 studies with individual patient randomization (5699 patients), most published before 2009, was an apparent prevention effect (log risk ratio −0.16; −0.26 to −0.06) whereas the summary ES from three cluster randomized studies with randomization of populations (18 335 patients), all published since 2009, showed zero prevention effect (log risk ratio 0; −0.24 to +0.26). Why the difference?

**Figure 3.**
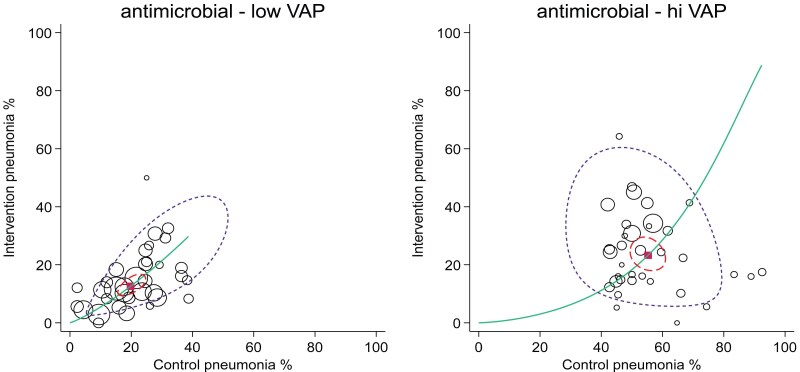
Summary receiver operating characteristic curves with 95% confidence limits (dotted inner ellipse) and 95% prediction limits (dotted outer ellipse) of pneumonia incidence among control and intervention groups (symbol size proportional to group size) from studies of antimicrobial-based pneumonia (VAP) prevention interventions with control group VAP proportions less than (left, *n* = 25) versus greater than (right, *n* = 25) 40%. These studies are drawn from four Cochrane reviews of studies in ICU patients. Also shown is the summary point (solid square) and the hierarchical summary receiver operating characteristic curve (solid curve). Note that in this adaptation of the summary receiver operating characteristic plot to visualize RCT data in the DTA framework, the intervention group incidence equates to ‘sensitivity’ and the control group incidence equals 1 minus ‘specificity’. The method and original data are listed in.^48^

Second, the pneumonia proportions are unusually high. The expert range for VAP proportion, as acknowledged in these SRs, is 5% to 40%. However, among these SRs, the VAP incidence exceeds 40% for the control groups of 30% and 50% of the chlorhexidine and TAP ± PPAP studies, respectively (Figure 2).^54–56^ These high pneumonia proportions, noted in the median event rates in the SR summary tables, originate from studies without major ROB concerns identified and remain unexplained. They become apparent only in an arms-based reanalysis of the VAP data from all 13 SRs (Figures 2 and 3). Surprisingly, the mean pneumonia proportions and 95% PIs of the TAP ± PPAP, chlorhexidine and non-antimicrobial intervention groups align closely not only with each other but also with the expert range and also align with the 95% PIs of the non-antibiotic control groups (Figure 2).

Likewise, other end points among control groups of decontamination studies are unusually high whereas those among intervention groups are not unusually low when compared with external benchmarks. The bacteraemia proportions among control groups of studies of TAP ± PPAP as summarized in another SR,^58^ are also unusually high.^59^ The mortality incidence, using even the conservative counts from Minozzi^47^ and even after adjusting for group mean age, among concurrent control groups of antimicrobial-based decontamination studies, is five percentage points higher than expected and showed greater dispersion, whereas that among the intervention groups is comparable to what is expected.^60^

Third, the pneumonia proportions among the chlorhexidine and TAP ± PPAP intervention groups are not unusually low with only two of 20 and three of 30, respectively, being <5% versus 10 of 115 among the non-antimicrobial intervention groups (Figure 2).

Fourth, there are major differences in numerator and denominator counts on which the summary ES estimates are based (Tables S2 and S3) with several differences exceeding 20% between counts in the Liberati^46^ versus Minozzi^47^ SRs. As noted in the SRs, these differences can be attributed to whether the counts were obtained from the studies as published (Minozzi^47^) or from the authors by personal communication (Liberati^46^). These differences presumably originate from assumptions in the early SDD studies wherein pneumonia rather than mortality was the primary end point of interest, and the TAP intervention was believed to be most effective towards preventing infection acquired after the first 4 days of application. On that basis, excluding the early counts to obtain per-protocol data towards pneumonia ES estimates was logical.^61^ However, the discrepancies are not acknowledged in the most recent SR, nor are they obvious to an inexpert reader, and excluding early mortality counts is problematic. Moreover, more recent studies of the TAP intervention use intention to treat data without excluding early counts.^57^

Finally, the most serious concern relates to whether the SUTVA assumption is reasonable in the ICU setting? The first study of TAP,^62^ and others,^57^ thought not and presumed that the results of TAP intervention would differ for CRT versus RCT studies. SUTVA has not been considered, let alone tested in these SRs. If spillover is possible, it is neither logical nor safe to use per-protocol data.

## The inference pivot

Valid causal inferences from the SRs of chlorhexidine and TAP ± PPAP pivot on the required assumptions, being that SUTVA is valid, there is no spillover, the ROB and heterogeneity are acceptable, and that the data as published are fit for the PICO research question, which relates to VAP prevention interventions targeted at individual patients. Guidelines for zero-pneumonia can build on these causal inferences only if the assumptions are deemed tenable. However, the unusually high VAP incidences, together with the other unacknowledged data discrepancies, cast doubt on the ‘certainty’ of the SR findings.

Does the ES from RCTs vary with underlying risk?^63^ Differences in underlying risk were found to explain the disparate results between the preclinical and clinical trials of anti-inflammatory agents for sepsis.^64^

The studies of chlorhexidine and TAP interventions that have control groups with unusually high VAP proportions might differ for inapparent reasons from those with VAP within the expected range. Recalculating the summary ES separately for subgroups of studies with control group VAP proportions greater than versus less than 40% for all antimicrobial (chlorhexidine and TAP ± PPAP) studies gives a summary ES (and 95% CI) of 0.26; 0.18–0.35 (*n* = 35) versus 0.59; 0.48–0.73 (*n* = 33), respectively (Figure 3) whereas the mean and 95% PI for the two strata of intervention groups in these studies, being 25; 9.2–52 and 13; 4.2–35, respectively, each align with the expected VAP range.^48^ This does not augur well for zero VAP.

However, when the RCT incidence data is benchmarked against incidence data observed outside the RCTs, such as the VAP range issued by expert opinion, the assumptions underlying the antimicrobial SRs are questionable. If the assumptions are deemed not tenable, are the inferences from these SRs ‘flawed beyond repair’?^3^ Moreover, for population-based initiatives, such as zero-Pneumonia, evaluation of these interventions within CRTs, wherein ICU populations are randomized, would be preferrable.

The causal inference pivots if viewed from a population perspective. Spillover, which is harmful, occurring in RCTs of topical antimicrobial interventions in the ICU setting would explain the unusually high VAP proportions in RCTs. Moreover, the absence of spillover in CRTs would explain the zero ES of decontamination interventions there. Of note, these two inferences, with benefit at the individual level versus harm at the population level, are not incompatible.

## Future

To remain relevant to evidence-based medicine, SRs will need to recognize and validate the key underlying assumptions. In developing guidelines for ‘pneumonia zero’, are the findings of SRs of various VAP prevention interventions safe? Are they useable? The case study previously raises a more general question for any SR. How can SRs best recognize and validate the underlying assumptions? Increasingly, this will require data from external sources relevant to the PICO research question and scrutiny of the study data versus external benchmarks if available.^65,66^

NMA is an emerging approach to deal with several of the challenges raised previously. NMA requires careful attention to ensure the comparator groups are truly comparable. The findings of an NMA for antibiotic prophylaxis in patients with cancer differed depending on how the comparator and control groups were grouped.^67^ NMA has additional assumptions, particularly that of transitivity, being that any effect modifiers are similarly distributed across the observed trial comparisons in the analysis network.^67–70^ One NMA example is that of antibiotic choice for the medical versus surgical management of acute appendicitis. This NMA enabled broad comparisons using data obtained from several RCTs.^68^ However, for this NMA, the RCT data were limited so observational studies were incorporated using a Bayesian approach.

Graphical displays, such as the caterpillar plot, are better than the traditional forest plot for visualizing data dispersion and the balance between potential outlier versus inlier study results towards the summary effect in SRs. There are >200 types of graphical display available.^65,66^ The L’abbe plot has long been used for this role but has deficiencies, especially where there are limited data.^30^ There are newer techniques to display the data that will help to identify outlier results.

Another emerging technique is that of the directed acyclic graph to objectively depict the assumptions and potential confounders that underlie the data generating mechanisms across different studies.^71^ A recent example has considered the application of directed acyclic graphs for assessing the impact of time varying confounders on the relationship between antibiotic de-escalation and patient mortality.^72^

Another tool that may emerge is structural equation modelling, which potentially might enable univariate and multivariate meta-analysis to enable comparisons of several treatments.^73^

## Conclusion

SRs have evolved over the past 25 years. SRs are powerful tools for summarizing a diverse and sometimes controversial literature. The strengths and weaknesses of SRs critically depend on the validity of their assumptions. As with any other research tool, the safety of SR inferences and their application to evidence-based medicine need care. Increasingly, SRs will need to account for the primary study data provenance beyond any ROB assessments. Methods to visualize the data in relation to external benchmarks, if available, will help ensure this.

## Supplementary Material

dkaf282_Supplementary_Data

## Data sharing statement

The data that support the findings of this study are contained within the on line Supplementary Material and related publications.

## Supplementary data

Tables S1 to S3 are available as Supplementary data at *JAC* Online.

## References

[1] Higgins JPT, Thomas J, Chandler J et al eds. Cochrane Handbook for Systematic Reviews of Interventions version 6.5 (updated August 2024). Cochrane, 2024. www.cochrane.org/handbook.

[2] Leibovici L, Reeves D. Systematic reviews and meta-analyses in the *Journal of Antimicrobial Chemotherapy*. J Antimicrob Chemother 2005; 56: 803–4. 10.1093/jac/dki340 16166177

[3] Ioannidis JP . The mass production of redundant, misleading, and conflicted systematic reviews and meta-analyses. Milbank Q 2016; 94: 485–514. 10.1111/1468-0009.12210 27620683 PMC5020151

[4] Møller MH, Ioannidis JP, Darmon M. Are systematic reviews and meta-analyses still useful research? We are not sure. Intensive Care Med 2018; 44: 518–20. 10.1007/s00134-017-5039-y 29663048

[5] Chevret S, Ferguson ND, Bellomo R. Are systematic reviews and meta-analyses still useful research? No. Intensive Care Med 2018; 44: 515–7. 10.1007/s00134-018-5066-3 29663047

[6] Page MJ, McKenzie JE, Bossuyt PM et al The PRISMA 2020 statement: an updated guideline for reporting systematic reviews. BMJ 2021; 372: n71. 10.1136/bmj.n71 33782057 PMC8005924

[7] Thomas C, Stevenson M, Riley TV. Antibiotics and hospital-acquired *Clostridium difficile*-associated diarrhoea: a systematic review. J Antimicrob Chemother 2003; 51: 1339–50. 10.1093/jac/dkg254 12746372

[8] Schwaber MJ, Carmeli Y. Mortality and delay in effective therapy associated with extended-spectrum β-lactamase production in Enterobacteriaceae bacteraemia: a systematic review and meta-analysis. J Antimicrob Chemother 2007; 60: 913–20. 10.1093/jac/dkm318 17848376

[9] Wang Y, Bahar MA, Jansen AM et al Improving antibacterial prescribing safety in the management of COPD exacerbations: systematic review of observational and clinical studies on potential drug interactions associated with frequently prescribed antibacterials among COPD patients. J Antimicrob Chemother 2019; 74: 2848–64. 10.1093/jac/dkz221 31127283 PMC6814093

[10] Li AM, Gomersall CD, Choi G et al A systematic review of antibiotic dosing regimens for septic patients receiving continuous renal replacement therapy: do current studies supply sufficient data? J Antimicrob Chemother 2009; 64: 929–37. 10.1093/jac/dkp302 19706668

[11] Brown EM . Optimal designs of clinical studies in the context of their inclusion in systematic reviews. J Antimicrob Chemother 2021; 76: 2498–500. 10.1093/jac/dkab227 34179970

[12] Martin A, Panaiotov S, Portaels F et al The nitrate reductase assay for the rapid detection of isoniazid and rifampicin resistance in *Mycobacterium tuberculosis*: a systematic review and meta-analysis. J Antimicrob Chemother 2008; 62: 56–64. 10.1093/jac/dkn139 18407918

[13] Martin A, Portaels F, Palomino JC. Colorimetric redox-indicator methods for the rapid detection of multidrug resistance in *Mycobacterium tuberculosis*: a systematic review and meta-analysis. J Antimicrob Chemother 2007; 59: 175–83. 10.1093/jac/dkl477 17135182

[14] Hurley J . Meta-analysis of clinical studies of diagnostic tests: developments in how the receiver operating characteristic “works”. Arch Pathol & Lab Med 2011; 135: 1585–90. 10.5858/arpa.2011-0016-SO 22129189

[15] Ghahramani A, Naghadian Moghaddam MM, Kianparsa J et al Overall status of carbapenem resistance among clinical isolates of *Acinetobacter baumannii*: a systematic review and meta-analysis. J Antimicrob Chemother 2024; 79: 3264–80. 10.1093/jac/dkae358 39392464

[16] Capodici A, Salussolia A, Sanmarchi F et al Biased, wrong and counterfeited evidences published during the COVID-19 pandemic, a systematic review of retracted COVID-19 papers. Qual Quant 2023; 57: 4881–913. 10.1007/s11135-022-01587-3 PMC9707851 36466994

[17] Hill A, Mirchandani M, Ellis L et al Ivermectin for the prevention of COVID-19: addressing potential bias and medical fraud. J Antimicrob Chemother 2022; 77: 1413–6. 10.1093/jac/dkac052 35190831 PMC9326581

[18] Lawrence JM, Meyerowitz-Katz G, Heathers JA et al The lesson of ivermectin: meta-analyses based on summary data alone are inherently unreliable. Nat Med 2021; 27: 1853–4. 10.1038/s41591-021-01535-y 34552263

[19] Cong W, Cheng HY, Stuart B et al Prevalence of antibiotic prescribing in COVID-19 patients in China and other low-and middle-income countries during the pandemic (December 2019—March 2021): a systematic review and meta-analysis. J Antimicrob Chemother 2023; 78: 2787–94. 10.1093/jac/dkad302 37883697 PMC10689912

[20] Kalil AC . Is cefepime safe for clinical use? A Bayesian viewpoint. J Antimicrob Chemother 2011; 66: 1207–9. 10.1093/jac/dkr138 21471137

[21] Yarnell CJ, Abrams D, Baldwin MR et al Clinical trials in critical care: can a Bayesian approach enhance clinical and scientific decision making? Lancet Respir Med 2021; 9: 207–16. 10.1016/S2213-2600(20)30471-9 33227237 PMC8439199

[22] Hurley JC . How the cluster randomized trial “works”. Clin Infect Dis 2020; 70: 341–6. 10.1093/cid/ciz554 31260511

[23] Oldenburg CE, Arzika AM, Amza A et al Mass azithromycin distribution to prevent childhood mortality: a pooled analysis of cluster-randomized trials. Am J Trop Med Hygiene 2019; 100: 691. 10.4269/ajtmh.18-0846 PMC6402901 30608051

[24] Leibovici L, Soares-Weiser K, Paul M et al Considering resistance in systematic reviews of antibiotic treatment. J Antimicrob Chemother 2003; 52: 564–71. 10.1093/jac/dkg409 12951336

[25] Clemens J, Shin S, Ali M. New approaches to the assessment of vaccine herd protection in clinical trials. Lancet Infect Dis 2011; 11: 482–7. 10.1016/S1473-3099(10)70318-2 21616458

[26] Reeves BC, Deeks JJ, Higgins JPT et al Chapter 24: including non-randomized studies on intervention effects [last updated October 2019]. In: Higgins JPT, Thomas J, Chandler J et al, eds. Cochrane Handbook for Systematic Reviews of Interventions Version 6.5. Cochrane, 2024. cochrane.org/handbook.

[27] Van Nieuwenhoven CA, Buskens E, Van Tiel FH et al Relationship between methodological trial quality and the effects of selective digestive decontamination on pneumonia and mortality in critically ill patients. JAMA 2001; 286: 335–40. 10.1001/jama.286.3.335 11466100

[28] Borenstein M, Higgins JP, Hedges LV et al Basics of meta-analysis: I2 is not an absolute measure of heterogeneity. Res synth method 2017; 8: 5–18. 10.1002/jrsm.1230 28058794

[29] Borenstein M . In a meta-analysis, the *I*-squared statistic does not tell US how much the effect size varies. J Clin Epidemiol 2022; 152: 281–4. 10.1016/j.jclinepi.2022.10.003 36223816

[30] Song F . Exploring heterogeneity in meta-analysis: is the L'Abbe plot useful? J Clin Epidemiol 1999; 52: 725–30. 10.1016/S0895-4356(99)00066-9 10465316

[31] IntHout J, Ioannidis JP, Rovers MM et al Plea for routinely presenting prediction intervals in meta-analysis. BMJ Open 2016; 6: e010247. 10.1136/bmjopen-2015-010247 PMC4947751 27406637

[32] Graham PL, Moran JL. Robust meta-analytic conclusions mandate the provision of prediction intervals in meta-analysis summaries. J Clin Epidemiol 2012; 65: 503–10. 10.1016/j.jclinepi.2011.09.012 22265586

[33] Pan K, Li R, Li Y et al Vancomycin combined with piperacillin/tazobactam increases the risk of acute kidney injury compared with vancomycin plus other anti-pseudomonal beta-lactams: a systematic review and network meta-analysis. J Antimicrob Chemother 2025; 80:47–58. 10.1093/jac/dkae410. 39533846

[34] Hong H, Chu H, Zhang J et al Rejoinder to the discussion of “a Bayesian missing data framework for generalized multiple outcome mixed treatment comparisons,” by S. Dias and AE Ades. Res Synth Method 2016; 7: 29–33. 10.1002/jrsm.1186 PMC4779393 26461816

[35] Toews I, George AT, Peter JV et al Interventions for preventing upper gastrointestinal bleeding in people admitted to intensive care units. Cochrane Database Syst Rev 2018; 6: CD008687. 10.1002/14651858.CD008687.pub2 29862492 PMC6513395

[36] Lewis SR, Schofield-Robinson OJ, Alderson P et al Enteral versus parenteral nutrition and enteral versus a combination of enteral and parenteral nutrition for adults in the intensive care unit. Cochrane Database Syst Rev 2018; 6: CD012276. 10.1002/14651858.CD012276.pub2 29883514 PMC6353207

[37] Padilla PF, Martínez G, Vernooij RW et al Early enteral nutrition (within 48 hours) versus delayed enteral nutrition (after 48 hours) with or without supplemental parenteral nutrition in critically ill adults. Cochrane Database Syst Rev 2019; 2019: CD012340. 10.1002/14651858.CD012340.pub2 31684690 PMC6820694

[38] Alkhawaja S, Martin C, Butler RJ et al Post-pyloric versus gastric tube feeding for preventing pneumonia and improving nutritional outcomes in critically ill adults. Cochrane Database Syst Rev 2015; 2015: CD008875. 10.1002/14651858.CD008875.pub2 26241698 PMC6516803

[39] Solà I, Benito S. Closed tracheal suction systems versus open tracheal suction systems for mechanically ventilated adult patients. Cochrane Database Syst Rev 2007; 2007: CD004581. 10.1002/14651858.CD004581.pub2 17943823 PMC6517217

[40] Gillies D, Todd DA, Foster JP et al Heat and moisture exchangers versus heated humidifiers for mechanically ventilated adults and children. Cochrane Database Syst Rev 2017; 9: CD004711. 10.1002/14651858.CD004711.pub3 28905374 PMC6483749

[41] Wang L, Li X, Yang Z et al Semi-recumbent position versus supine position for the prevention of ventilator-associated pneumonia in adults requiring mechanical ventilation. Cochrane Database Syst Rev 2016; 2016: CD009946. 10.1002/14651858.CD009946.pub2 26743945 PMC7016937

[42] Tokmaji G, Vermeulen H, Müller MCA et al Silver-coated endotracheal tubes for prevention of ventilator-associated pneumonia in critically ill patients. Cochrane Database Syst Rev 2015; 2015: CD009201. 10.1002/14651858.CD009201.pub2 26266942 PMC6517140

[43] Bo L, Li J, Tao T et al Probiotics for preventing ventilator-associated pneumonia. Cochrane Database Syst Rev 2014; 2014: CD009066. 10.1002/14651858.CD009066.pub2 25344083 PMC4283465

[44] Hua F, Xie H, Worthington HV et al Oral hygiene care for critically ill patients to prevent ventilator-associated pneumonia. Cochrane Database Syst Rev 2016; 2016: CD008367. 10.1002/14651858.CD008367.pub3. PMC6460950 27778318

[45] Zhao T, Wu X, Zhang Q et al Oral hygiene care for critically ill patients to prevent ventilator-associated pneumonia. Cochrane Database Syst Rev 2020; 2020: CD008367. 10.1002/14651858.CD008367.pub4. PMC8111488 33368159

[46] Liberati A, D’Amico R, Pifferi S et al Antibiotic prophylaxis to reduce respiratory tract infections and mortality in adults receiving intensive care. Cochrane Database Syst Rev 2009; 2009: CD000022. 10.1002/14651858.CD000022.pub3 14973945

[47] Minozzi S, Pieri S, Brazzi L et al Topical antibiotic prophylaxis to reduce respiratory tract infections and mortality in adults receiving mechanical ventilation. Cochrane Database Syst Rev 2021; 2021: CD000022. 10.1002/14651858.CD000022.pub4. PMC8094382 33481250

[48] Hurley JC . Visualizing and diagnosing spillover within randomized concurrent controlled trials through the application of diagnostic test assessment methods. BMC Med Res Methodol 2024; 24: 182. 10.1186/s12874-024-02296-1 39152400 PMC11328391

[49] Álvarez-Lerma F, Palomar-Martínez M, Sánchez-García M et al Prevention of ventilator-associated pneumonia: the multimodal approach of the Spanish ICU “Pneumonia Zero” program. Crit Care Med 2018; 46: 181–8. 10.1097/CCM.0000000000002736 29023261 PMC5770104

[50] Hurley JC . Indirect (herd) effects of topical antibiotic prophylaxis and oral care versus non-antimicrobial methods on mortality among ICU patients. Realigning Cochrane review data to emulate a three-tier cluster randomized trial. BMJ Open 2023; 13: e064256. 10.1136/bmjopen-2022-064256 PMC10689355 38035749

[51] Hurley JC . Length of intensive care unit stay and the apparent efficacy of antimicrobial-based versus non-antimicrobial-based ventilator pneumonia prevention interventions within the Cochrane Review Database. J Hosp Infect 2023; 140: 46–53. 10.1016/j.jhin.2023.07.018 37544366

[52] Hurley J . Estimating the herd effects of anti-microbial-based decontamination (ABD) interventions on intensive care unit (ICU) acquired bloodstream infections: a deductive meta-analysis. BMJ Open 2024; 14: e092030. 10.1136/bmjopen-2024-092030 PMC11580273 39572099

[53] Hurley JC . Estimating the herd effects of antimicrobial prevention interventions on ventilator-associated pneumonia within ICU populations: a cluster randomized trial emulation using data from Cochrane reviews. J Antimicrob Chemother 2025; 80: 1047–58. 10.1093/jac/dkaf033 39928420 PMC11962382

[54] American Thoracic Society . Infectious Diseases Society of America: guidelines for the management of adults with hospital-acquired, ventilator-associated, and healthcare-associated pneumonia. Am J Respir Crit Care Med 2005; 171: 388–416. 10.1164/rccm.200405-644ST 15699079

[55] Chevret S, Hemmer M, Carlet J et al European Cooperative Group on Nosocomial Pneumonia. Incidence and risk factors of pneumonia acquired in intensive care units: results from a multicenter prospective study on 996 patients. Intensive Care Med 1993; 19: 256–64. 10.1007/BF01690545 8408934

[56] Chastre J, Fagon JY. Ventilator-associated pneumonia. Am J Respir Crit Care Med 2002; 165: 867–903. 10.1164/ajrccm.165.7.2105078 11934711

[57] de Smet AM, Kluytmans JA, Cooper BS et al Decontamination of the digestive tract and oropharynx in ICU patients. N Engl J Med 2009; 360: 20–31. 10.1056/NEJMoa0800394 19118302

[58] Hammond NE, Myburgh J, Seppelt I et al Association between selective decontamination of the digestive tract and in-hospital mortality in intensive care unit patients receiving mechanical ventilation: a systematic review and meta-analysis. JAMA 2022; 328: 1922–34. 10.1001/jama.2022.19709 36286098 PMC9607997

[59] Hurley JC . Selective digestive decontamination-Con. Intensive Care Med 2023; 49: 982–3. 10.1007/s00134-023-07146-0 37386314

[60] Hurley JC . Trends in ICU mortality and underlying risk over three decades among mechanically ventilated patients. A group level analysis of cohorts from infection prevention studies. Annals Intensive Care 2023; 13: 62. 10.1186/s13613-023-01159-0 PMC10335996 37432605

[61] Smith VA, Coffman CJ, Hudgens MG. Interpreting the results of intention-to-treat, per-protocol, and as-treated analyses of clinical trials. JAMA 2021; 326: 433–4. 10.1001/jama.2021.2825 34342631 PMC8985703

[62] Stoutenbeek CP, Van Saene HK, Miranda DR et al The effect of selective decontamination of the digestive tract on colonisation and infection rate in multiple trauma patients. Intensive Care Med 1984; 10: 185–92. 10.1007/BF00259435 6470306

[63] Moran JL, Graham PL. Risk related therapy in meta-analyses of critical care interventions: Bayesian meta-regression analysis. J Crit Care 2019; 53: 114–9. 10.1016/j.jcrc.2019.06.003 31228761

[64] Eichacker PQ, Parent C, Kalil A et al Risk and the efficacy of agents: retrospective and confirmatory studies of sepsis. Am J Respir Crit Care Med 2002; 166: 1197–205. 10.1164/rccm.200204-302OC 12403688

[65] Kossmeier M, Tran US, Voracek M. Charting the landscape of graphical displays for meta-analysis and systematic reviews: a comprehensive review, taxonomy, and feature analysis. BMC Med Res Methodol 2020; 20: 1–24. 10.1186/s12874-020-0911-9 PMC7006175 32028897

[66] Hurley JC . Forrest plots or caterpillar plots? J Clin Epidemiol 2020; 121: 109–10. 10.1016/j.jclinepi.2020.01.017 32014534

[67] Martinez JP, Robinson PD, Phillips B et al Conventional compared to network meta-analysis to evaluate antibiotic prophylaxis in patients with cancer and haematopoietic stem cell transplantation recipients. BMJ Evid Based Med 2021; 26: 320–6. 10.1136/bmjebm-2020-111362 32868288

[68] Wang CH, Yang CC, Hsu WT et al Optimal initial antibiotic regimen for the treatment of acute appendicitis: a systematic review and network meta-analysis with surgical intervention as the common comparator. J Antimicrob Chemother 2021; 76: 1666–75. 10.1093/jac/dkab074 33792691

[69] Kengkla K, Kongpakwattana K, Saokaew S et al Comparative efficacy and safety of treatment options for MDR and XDR *Acinetobacter baumannii* infections: a systematic review and network meta-analysis. J Antimicrob Chemother 2018; 73: 22–32. 10.1093/jac/dkx368 29069421

[70] Ordóñez-Mena JM, McCarthy ND, Fanshawe TR. Comparative efficacy of drugs for treating giardiasis: a systematic update of the literature and network meta-analysis of randomized clinical trials. J Antimicrob Chemother 2018; 73: 596–606. 10.1093/jac/dkx430 29186570 PMC5890742

[71] Dijk SW, Caulley LM, Hunink M et al From complexity to clarity: how directed acyclic graphs enhance the study design of systematic reviews and meta-analyses. Eur J Epidemiol 2024; 39: 27–33. 10.1007/s10654-023-01042-z 37650986 PMC10811040

[72] Van Heijl I, Schweitzer VA, Van Der Linden PD et al Impact of antimicrobial de-escalation on mortality: a literature review of study methodology and recommendations for observational studies. Expert Rev Anti Infect Ther 2020; 18: 405–13. 10.1080/14787210.2020.1743683 32178545

[73] Tu YK, Wu YC. Using structural equation modeling for network meta-analysis. BMC Med Res Methodol 2017; 17: 1–9. 10.1186/s12874-017-0390-9 28709406 PMC5512972

