# Correction to: Systematic reviews and the *Journal of Antimicrobial Chemotherapy;* past, present and future. A systematic reappraisal

**DOI:** 10.1093/jac/dkaf403

**Published:** 2025-10-24

This is a correction to: James C Hurley, Systematic reviews and the *Journal of Antimicrobial Chemotherapy;* past, present and future. A systematic reappraisal, *Journal of Antimicrobial Chemotherapy*, 2025; https://doi.org/10.1093/jac/dkaf282

The following changes have been made to the originally published manuscript.

Table 1 column headers and the table's footnotes have been emended to read:

**Table 1.** Summary results from 13 systematic reviews of interventions to prevent pneumonia and mortality in ICU patients

SR^a^	Patients	Studies	Median event rate (per 1000 patients)		Effect size				ITT/PP
	(n)	(n)	Control	Intervention	RR	95% CI	I^2^	Certainty	
Pneumonia prevention									
Non-antimicrobial									
Composite^35–43^	24 594	122	75–314	48–268	0.82	0.71–0.93	50	Low to moderate	ITT
Chlorhexidine									
Hua^44^	2451	18	243	180	0.75	0.62–0.91	35	High	ITT
Zhao^45^	1206	13^b^	261	175	0.67	0.47–0.97	66	Moderate	ITT
TAP + PPAP^c^									
Liberati^46^	3024	16	NR	NR	0.28	0.2–0.38	56	NS^d^	ITT
Minozzi^47^	2951	17	417	179	0.43	0.35–0.53	55	Moderate	PP
TAP alone^c^									
Liberati^46^	1735	12	NR	NR	0.34	0.21–0.55	70	NS^d^	ITT
Minozzi^47^	1848	13	324	162	0.5	0.36–0.69	66	Low	PP
Mortality prevention									
Chlorhexidine									
Hua^44^	2014	14	222	242	1.09	0.96–1.23	0	Moderate	ITT
Zhao^45^	944	9^b^	190	247	1.03	0.8–1.33	0	Moderate	ITT
TAP + PPAP^c^									
Liberati^46^	4075	17	NR	NR	0.75	0.65–0.87	0	NS^d^	ITT
Minozzi^47^	5290	18	303	225	0.84	0.73–0.96	40	High	PP
TAP alone^c^									
Liberati^46^	1783	13	NR	NR	0.97	0.79–1.2	0	NS^d^	ITT
Minozzi^47^	3274	15	305	296	0.97	0.87–1.07	0	Moderate	PP

^a^Nine SRs of pneumonia prevention in ICU patients using gastrointestinal,^35^ feeding,^36–38^ airway^39–42^ and probiotic^43^ based interventions are presented here as a composite. None of these interventions achieved significant mortality prevention. The median event rate is a range among the nine SRs and the composite ES estimate is from.^48^

^b^Eight studies included in Hua meta-analysis were excluded from corresponding meta-analysis in Zhao^45^ (see Table S1).

^c^Results in many TAP studies were published as per protocol (PP) and used by Minozzi^47^ whereas the intention to treat (ITT) data were obtained as personal communications by Liberati from study authors (Tables S2 and S3).

^d^For Liberati,^46^ certainty is not stated (NS) except that publication bias was considered unlikely on the basis of the appearance of the funnel plot for the VAP prevention ES distribution.

instead of:

**Table 1.** Summary results from 13 SRs of interventions to prevent pneumonia and mortality in ICU patients

SR^a^	Patients		Studies		Median event rate (per 1000 patients)		Effect size		ITT/PP
	(*n*)	(*n*)	Control	Intervention	RR	95% CI	*I* ^2^ (%)	Certainty	
Pneumonia prevention									
Non-antimicrobial									
Composite^35–43^	24 594	122	75–314	48–268	0.82	0.71–0.93	50	Low to moderate	ITT
Chlorhexidine									
Hua^44^	2451	18	243	180	0.75	0.62–0.91	35	High	ITT
Zhao^45^	1206	13^b^	261	175	0.67	0.47–0.97	66	Moderate	ITT
TAP + PPAP^c^									
Liberati^46^	3024	16	NR	NR	0.28	0.2–0.38	56	NS^d^	ITT
Minozzi^47^	2951	17	417	179	0.43	0.35–0.53	55	Moderate	PP
TAP alone^c^									
Liberati^46^	1735	12	NR	NR	0.34	0.21–0.55	70	NS^d^	ITT
Minozzi^47^	1848	13	324	162	0.5	0.36–0.69	66	Low	PP
Mortality prevention									
Chlorhexidine									
Hua^44^	2014	14	222	242	1.09	0.96–1.23	0	Moderate	ITT
Zhao^45^	944	9^b^	190	247	1.03	0.8–1.33	0	Moderate	ITT
TAP + PPAP^c^									
Liberati^46^	4075	17	NR	NR	0.75	0.65–0.87	0	NS^d^	ITT
Minozzi^47^	5290	18	303	225	0.84	0.73–0.96	40	High	PP
TAP alone^c^									
Liberati^46^	1783	13	NR	NR	0.97	0.79–1.2	0	NS^d^	ITT
Minozzi^47^	3274	15	305	296	0.97	0.87–1.07	0	Moderate	PP

^a^Nine SRs of pneumonia prevention in ICU patients using gastrointestinal,^35^ feeding,^36–38^ airway^39–42^ and probiotic^43^ based interventions are presented here as a composite. None of these interventions achieved significant mortality prevention. The median event rate is a range among the nine SRs and the composite ES estimate is from.^48^

^b^Eight studies included in Hua meta-analysis were excluded from corresponding meta-analysis in Zhao^45^ (see Table S1).

^c^Results in many TAP studies were published as per protocol (PP) and used by Minozzi^47^ whereas the intention to treat (ITT) data were obtained as personal communications by Liberati from study authors (Tables S2 and S3).

^d^For Liberati,^46^ certainty is not stated (NS) except that publication bias was considered unlikely on the basis of the appearance of the funnel plot for the VAP prevention ES distribution.

The supplementary data file was published without coloured highlights to indicate differences in data: the highlighted text has been restored in the file now re-supplied with the article.

