# Supplementary material for: Systematic reviews and the Journal of Antimicrobial Chemotherapy; past, present and future. A systematic reappraisal
Source: J Antimicrob Chemother. 2025 Aug 21;80(10):2587–96. doi: 10.1093/jac/dkaf282 (PMC12494136; doi:10.1093/jac/dkaf282)
Supplement: dkaf282_Supplementary_Data [file dkaf282_supplementary_data.docx]

**Systematic reviews and *The*** ***Journal of Antimicrobial Chemotherapy;* past, present and future. A systematic reappraisal – Supplementary data**

| Contents | page |
| --- | --- |
| of topical antibiotics used to prevent ICU infections |  |
| Table s1; Data from Cochrane reviews of topical Chlorhexidine | p 2 – 4 |
| Table s2: Data from Cochrane reviews of TAP alone or TAP + PPAP versus PPAP alone (duplex) | p 5 – 9 |
| Table s3: Data from Cochrane reviews of TAP + PPAP | p 9-11 |
| References | p 12 – 15 |

Abbreviations: TAP = Topical antibiotic prophylaxis; PPAP = protocolized parenteral antibiotic prophylaxis; ITT = intention to treat; PP = per protocol

Data in Tables s1-s3 from;

1. Hua F, Xie H, Worthington HV, Furness S, Zhang Q, Li C. Oral hygiene care for critically ill patients to prevent ventilator-associated pneumonia. Cochrane Database of Systematic Reviews 2016, Issue 10. Art. No.: CD008367.
2. Zhao T, Wu X, Zhang Q, Li C, Worthington HV, Hua F. Oral hygiene care for critically ill patients to prevent ventilator-associated pneumonia. Cochrane Database of Systematic Reviews 2020, Issue 12. Art. No.: CD008367.
3. D'Amico R, Pifferi S, Leonetti C, Torri V, Tinazzi A, Liberati A. Effectiveness of antibiotic prophylaxis in critically ill adult patients: systematic review of randomised controlled trials. *BMJ*. 1998;316(7140):1275-85.
4. Liberati A, D’Amico R, Pifferi S, Torri V, Brazzi L, Parmelli E. Antibiotic prophylaxis to reduce respiratory tract infections and mortality in adults receiving intensive care. *Cochrane Database of Systematic Reviews* 2009, Issue 4. Art. No.: CD000022.
5. Minozzi S, Pieri S, Brazzi L, Pecoraro V, Montrucchio G, D'Amico R. Topical antibiotic prophylaxis to reduce respiratory tract infections and mortality in adults receiving mechanical ventilation. *Cochrane Database of Systematic Reviews* 2021, Issue 1. Art. No.: CD000022.

**Table S1: Pneumonia & mortality data Cochrane reviews of Chlorhexidine**

|  |  |  |  | Hua 2017 | | | | LOS | | Zhao 2020 | | | |
| --- | --- | --- | --- | --- | --- | --- | --- | --- | --- | --- | --- | --- | --- |
|  |  |  |  | intervention | | control | |  |  | intervention | | control | |
| author | Year | Ref | Notes | n | N | n | N |  |  | n | N | n | N |
| Pneumonia | |  |  |  |  |  |  |  |  |  |  |  |  |
| Bellissimo-Rodrigues | 2009 | 1 |  | 16 | 64 | 17 | 69 | 10 | 10 | 16 | 64 | 17 | 69 |
| Berry | 2011 | 2 | a | 4 | 71 | 1 | 78 |  |  | 4 | 33 | 1 | 43 |
| Cabov | 2010 | 3 |  | 1 | 17 | 6 | 23 |  |  | 1 | 17 | 6 | 23 |
| Chen | 2008 | 4 | b | 16 | 60 | 28 | 60 |  |  |  |  |  |  |
| De Riso | 1996 | 5 | c | 5 | 173 | 17 | 180 |  |  |  |  |  |  |
| Fourrier'00 | 2000 | 6 | d | 5 | 30 | 14 | 28 | 24 | 18 |  |  |  |  |
| Fourrier'05 | 2005 | 7 | e | 13 | 114 | 12 | 114 | 13 | 14 |  |  |  |  |
| Fu | 2019 | 8 | f |  |  |  |  | 10 | 7 | 7 | 40 | 37 | 40 |
| Grap | 2011 | 9 |  | 7 | 21 | 10 | 18 |  |  | 7 | 21 | 10 | 18 |
| Jacomo | 2011 | 10 | g | 16 | 87 | 11 | 73 |  |  |  |  |  |  |
| Koeman-Ch | 2006 | 11 | h | 13 | 127 | 23 | 130 | 12.5 | 13.8 | 13 | 127 | 23 | 130 |
| Kusahara | 2012 | 12 |  | 15 | 46 | 16 | 50 | 11 | 16 | 15 | 46 | 16 | 50 |
| Meidani | 2018 | 13 | i |  |  |  |  |  |  | 6 | 50 | 15 | 50 |
| Meinberg | 2012 | 14 |  | 18 | 28 | 11 | 24 |  |  | 18 | 28 | 11 | 24 |
| Munro | 2009 | 15 | j | 38 | 92 | 55 | 100 |  |  |  |  |  |  |
| Özçaka | 2012 | 16 |  | 12 | 29 | 22 | 32 | 15 | 12 | 12 | 29 | 22 | 32 |
| Panchabhai | 2009 | 62 | k | 14 | 88 | 15 | 83 |  |  |  |  |  |  |
| Scannapieco | 2009 | 17 |  | 14 | 97 | 12 | 49 |  |  | 14 | 97 | 12 | 49 |
| Sebastian | 2012 | 18 | l | 12 | 41 | 14 | 45 |  |  |  |  |  |  |
| Tantipong | 2008 | 19 |  | 5 | 58 | 10 | 52 |  |  | 5 | 58 | 10 | 52 |
| Tuon | 2017 | 20 | m |  |  |  |  |  |  | 4 | 8 | 2 | 8 |

Table S1: Pneumonia & mortality data Cochrane reviews of Chlorhexidine (continued)

|  |  |  |  | Hua 2017 | | | |  | Zhao 2020 | | | |
| --- | --- | --- | --- | --- | --- | --- | --- | --- | --- | --- | --- | --- |
|  |  |  |  | intervention | | control | |  | intervention | | control | |
| author | Year | Ref | Notes | n | N | n | N |  | n | N | n | N |
| Mortality | |  |  |  |  |  |  |  |  |  |  |  |
| Bellissimo-Rodrigues | 2009 | 1 |  | 34 | 64 | 32 | 69 |  | 34 | 64 | 32 | 69 |
| Cabov | 2010 | 3 |  | 0 | 17 | 0 | 23 |  | 0 | 17 | 0 | 23 |
| de Riso | 1996 | 5 | c | 2 | 173 | 10 | 180 |  |  |  |  |  |
| Fourrier'00 | 2000 | 6 | d | 3 | 30 | 7 | 30 |  |  |  |  |  |
| Fourrier'05 | 2005 | 7 | e | 31 | 114 | 24 | 114 |  |  |  |  |  |
| Fu | 2019 | 8 | f |  |  |  |  |  | 3 | 40 | 7 | 40 |
| Jacomo | 2011 | 10 | g | 5 | 87 | 5 | 73 |  |  |  |  |  |
| Koeman-Ch | 2006 | 11 | h |  |  |  |  |  | 95 | 255 | 38 | 130 |
| Kusahara | 2012 | 12 |  | 8 | 46 | 12 | 50 |  | 8 | 46 | 12 | 50 |
| Meidani | 2018 | 13 | i |  |  |  |  |  | 4 | 50 | 5 | 50 |
| Meinberg | 2012 | 14 |  | 13 | 28 | 9 | 24 |  | 13 | 28 | 9 | 24 |
| Munro | 2009 | 15 | j | 12 | 48 | 10 | 49 |  |  |  |  |  |
| Özçaka | 2012 | 16 |  | 17 | 29 | 19 | 32 |  | 17 | 29 | 19 | 32 |
| Panchabhai | 2009 | 62 | k | 64 | 88 | 51 | 83 |  |  |  |  |  |
| Scannapieco | 2009 | 17 |  | 16 | 116 | 8 | 59 |  | 16 | 116 | 8 | 59 |
| Sebastian | 2012 | 18 | l | 16 | 41 | 21 | 45 |  |  |  |  |  |
| Tantipong | 2008 | 19 |  | 36 | 102 | 37 | 105 |  | 36 | 102 | 37 | 105 |

Abbreviations; n = numerator count; N = denominator count

**Table S1 footnotes;**

1. Berry 2011; discrepancy in denominator counts relates to number randomized (Hua) versus number evaluated (Zhao).
2. Chen 2008; CHX versus saline: Excluded from Zhao 2020 systematic review as the intervention group received "routine oral care" but the control group did not
3. DeRiso 1996; Excluded from Zhao 2020 systematic review as unclear if all those who developed VAP had been on mechanical ventilation for at least 48 hours
4. Fourrier 2000; Excluded from Zhao 2020 systematic review as unclear if all those who developed VAP had been on mechanical ventilation for at least 48 hours
5. Fourrier 2005; Excluded from Zhao 2020 systematic review as unclear if all those who developed VAP had been on mechanical ventilation for at least 48 hours; some of the participants had pneumonia at baseline
6. Fu 2019; post-date the Hua 2017 systematic review.
7. Jacomo 2011; Excluded from Zhao 2020 systematic review as unclear how many patients have been on mechanical ventilation for at least 48 hours
8. Koeman 2006 is a three-arm study with topical chlorhexidine as the third arm. Mortality data recoverable from another systematic review [Melsen WG, Rovers MM, Groenwold RH, Bergmans DC, Camus C, Bauer TT, Hanisch EW, Klarin B, Koeman M, Krueger WA, Lacherade JC. Attributable mortality of ventilator-associated pneumonia: a meta-analysis of individual patient data from randomised prevention studies. Lancet infect Dis. 2013;13(8):665-71].
9. Meidani 2018; post-date the Hua 2017 systematic review.
10. Munro 2009; Excluded from Zhao 2020 systematic review as some of the patients had pneumonia at baseline
11. Panchabhai 2009; reclassified in Zhao 2020 systematic review as control group received oral care with potassium permanganate. Only per protocol data is available for those received the complete protocol. This study is excluded from the current analysis.
12. Sebastian 2012; Excluded from Zhao 2020 systematic review as most of the children admitted to ICU had pneumonia at baseline
13. Tuon 2017 post-date the Hua 2017 systematic review.

**Table S2: Pneumonia & mortality data for Cochrane reviews of TAP alone or duplex (= TAP + PPAP versus PPAP alone)**

|  |  |  |  | Liberati 2009 & D’amico 1998 | | | |  |  | Minozzi 2021 | | | |  |  |
| --- | --- | --- | --- | --- | --- | --- | --- | --- | --- | --- | --- | --- | --- | --- | --- |
|  |  |  |  | intervention | | control | | original | original | intervention | | control | | Minozzi 2021 | Minozzi 2021 |
| author | Year | Ref | Notes | n | N | n | N | LOS | LOS | n | N | n | N | LOS | LOS |
| Pneumonia (TAP alone) | |  |  |  |  |  |  |  |  |  |  |  |  |  |  |
| Bergmans | 2001 | 21 | a | 9 | 87 | 38 | 139 | 15 | 13 | 9 | 87 | 38 | 139 | 15 | 13 |
| Brun-Buisson | 1989 | 23 | c | 3 | 65 | 6 | 68 | 8 | 7 | 3 | 36 | 6 | 50 | 3.5 | 3.5 |
| Camus | 2005 | 24 | d | 53 | 130 | 53 | 126 | 16 | 16 | 53 | 130 | 53 | 126 | NR | NR |
| Gastinne | 1992 | 26 | g | 26 | 220 | 33 | 225 | 19 | 18 | 26 | 220 | 33 | 225 | 12 | 12 |
| Georges | 1994 | 27 |  | 4 | 31 | 15 | 33 | 16 | 16 | 4 | 31 | 15 | 33 | 33 | 33 |
| Koeman | 2006 | 28 | g |  |  |  |  |  |  | 16 | 128 | 23 | 130 | 13 | 13 |
| Korinek | 1993 | 29 | h | 20 | 96 | 37 | 95 | 25 | 27 | 20 | 63 | 37 | 60 | 26 | 26 |
| Pneumatikos | 2002 | 30 | i | 5 | 31 | 16 | 30 | 16 | 23 | 5 | 31 | 16 | 30 | 16 | 23 |
| Pugin | 1991 | 31 | j | 4 | 38 | 24 | 41 | 13.8 | 13.8 | 4 | 25 | 24 | 27 | 13.8 | 13.8 |
| Quinio | 1995 | 32 | k | 19 | 76 | 38 | 73 | 16 | 16 | 19 | 76 | 38 | 73 | 16 | 16 |
| Rodriguez-Roldan | 1990 | 33 | l | 1 | 14 | 11 | 17 | 12 | 10 | 1 | 13 | 11 | 15 | 13.5 | 13.5 |
| Unertl | 1987 | 34 | m | 1 | 19 | 9 | 20 | 18 | 23 | 1 | 19 | 9 | 20 | NR | NR |
| Wiener | 1995 | 35 | n | 8 | 30 | 8 | 31 | 11 | 11 | 8 | 30 | 8 | 31 | 11 | 11 |
| Pneumonia (duplex) | |  |  |  |  |  |  |  |  |  |  |  |  |  |  |
| Chaari | 2014 | 36 | o |  |  |  |  | 17 | 17 | 10 | 31 | 6 | 13 | NR | NR |
| Ferrer | 1994 | 37 | p | 7 | 51 | 11 | 50 | 15 | 14.3 | 7 | 51 | 11 | 50 | 7.5 | 7.5 |
| Hammond [1] | 1992 | 39 | r | 25 | 162 | 30 | 160 | 16 | 17 | 25 | 114 | 30 | 126 | 11 | 11 |
| Laggner | 1994 | 40 | s | 1 | 33 | 4 | 34 | 25 | 30 | 1 | 33 | 4 | 34 | 28.8 | 28.8 |
| Lingnau [5] | 1997 | 41 | t | 38 | 90 | 71 | 177 | 20 | 20 | 72 | 162 | 71 | 177 | 20 | 20 |
| Lingnau [5] | 1997 | 41 | t | 34 | 90 | 71 | 177 | 20 |  |  |  |  |  |  |  |
| Palomar | 1997 | 54 | u |  |  | 14 | 46 | 10 |  |  |  |  |  |  |  |
| Stoutenbeek | 1996 | 42 | v | 2 | 49 | 8 | 42 | 15 | 15 | 2 | 30 | 8 | 29 | 15 | 15 |

Table S2: Pneumonia & mortality data for Cochrane reviews of TAP alone alone or duplex (continued)

|  |  |  |  | Liberati 2009 & D’amico 1998 | | | |  |  | Minozzi 2021 | | | |  |  |
| --- | --- | --- | --- | --- | --- | --- | --- | --- | --- | --- | --- | --- | --- | --- | --- |
|  |  |  |  | intervention | | control | | original | original | intervention | | control | | Minozzi 2021 | Minozzi 2021 |
| author | Year | Ref | Notes | n | N | n | N | LOS | LOS | n | N | n | N | LOS | LOS |
| Mortality (TAP alone) | |  |  |  |  |  |  |  |  |  |  |  |  |  |  |
| Bergmans | 2001 | 21 | a | 30 | 87 | 59 | 139 | 15 | 13 | 30 | 87 | 59 | 139 | 15 | 13 |
| Beshey | 2014 | 22 | b |  |  |  |  | 15 | 15 | 0 | 50 | 0 | 25 | NR | NR |
| Brun-Buisson | 1989 | 23 | c | 14 | 65 | 15 | 68 | 8 | 7 | 8 | 36 | 12 | 50 | 3.5 | 3.5 |
| Camus | 2005 | 24 | d | 32 | 130 | 34 | 126 | 16 | 16 | 32 | 130 | 34 | 126 | NR | NR |
| Cerra | 1992 | 25 | e | 13 | 25 | 10 | 23 | 18 | 26 | 13 | 25 | 10 | 21 | NR | NR |
| de Smet SOD | 2009 | 61 | f |  |  |  |  |  |  | 235 | 765 | 254 | 799 | 8 | 7 |
| Gastinne | 1992 | 26 |  | 88 | 220 | 82 | 225 | 19 | 18 | 88 | 220 | 82 | 225 | 12 | 12 |
| Georges | 1994 | 27 |  | 3 | 31 | 5 | 33 | 16 | 16 | 3 | 31 | 5 | 33 | 33 | 33 |
| Koeman | 2006 | 28 | g |  |  |  |  |  |  |  |  |  |  |  |  |
| Korinek | 1993 | 29 | h | 22 | 96 | 17 | 95 | 25 | 27 | 5 | 63 | 4 | 60 | 26 | 26 |
| Pneumatikos | 2002 | 30 | i | 5 | 31 | 7 | 30 | 16 | 23 | 5 | 31 | 7 | 30 | 16 | 23 |
| Pugin | 1991 | 31 | j | 10 | 38 | 11 | 41 | 13.8 | 13.8 | 7 | 25 | 7 | 27 | 13.8 | 13.8 |
| Quinio | 1995 | 32 | k | 12 | 76 | 10 | 73 | 16 | 16 | 12 | 76 | 10 | 72 | 16 | 16 |
| Rodriguez-Roldan | 1990 | 33 | l | 5 | 14 | 7 | 17 | 12 | 10 | 4 | 13 | 5 | 15 | 13.5 | 13.5 |
| Unertl | 1987 | 34 | m | 5 | 19 | 6 | 20 | 18 | 23 | 5 | 19 | 6 | 20 | NR | NR |
| Wiener | 1995 | 35 | n | 11 | 30 | 15 | 31 | 11 | 11 | 11 | 30 | 15 | 31 | 11 | 11 |

Abbreviations; n = numerator count; N = denominator count

Table S2: Pneumonia & mortality data for Cochrane reviews of TAP alone alone or duplex (continued)

|  |  |  |  | Liberati 2009 & D’amico 1998 | | | |  |  | Minozzi 2021 | | | |  |  |
| --- | --- | --- | --- | --- | --- | --- | --- | --- | --- | --- | --- | --- | --- | --- | --- |
|  |  |  |  | intervention | | control | | original | original | intervention | | control | | Minozzi 2021 | Minozzi 2021 |
| author | Year | Ref | Notes | n | N | n | N | LOS | LOS | n | N | n | N | LOS | LOS |
| Mortality (duplex) |  |  |  |  |  |  |  |  |  |  |  |  |  |  |  |
| Chaari | 2014 | 36 | o |  |  |  |  | 17 | 17 | 8 | 31 | 6 | 13 | NR | NR |
| Ferrer | 1994 | 37 | p | 15 | 51 | 14 | 50 | 15 | 14.3 | 15 | 51 | 14 | 50 | 7.5 | 7.5 |
| Gaussorgues | 1991 | 38 | q | 29 | 59 | 29 | 59 | 16 | 19 | 29 | 59 | 29 | 59 | NR | NR |
| Hammond [1] | 1992 | 39 | r | 34 | 162 | 31 | 160 | 16 | 17 | 21 | 114 | 21 | 126 | 11 | 11 |
| Laggner | 1994 | 40 | s | 9 | 33 | 14 | 34 | 25 | 30 | 9 | 33 | 14 | 34 | 28.8 | 28.8 |
| Lingnau [5] | 1997 | 41 | t | 9 | 90 | 17 | 177 | 20 | 20 | 22 | 162 | 17 | 148 | 20 | 20 |
| Lingnau [5] | 1997 | 41 | t | 13 | 90 | 17 | 177 | 20 |  |  |  |  |  | 20 |  |
| Palomar | 1997 | 54 | u |  |  | 10 | 46 | 10 |  |  |  |  |  | 10 |  |
| Stoutenbeek | 1996 | 42 | v | 2 | 49 | 8 | 42 | 15 | 15 | 2 | 30 | 8 | 29 | 15 | 15 |

Abbreviations; n = numerator count; N = denominator count

**Table S2 footnotes;**

1. Bergmans 2001; 19 patients excluded because they did not fulfil inclusion criteria. This is a three-arm study with the two control groups (concurrent and non-concurrent) aggregated in the analysis of both reviews.
2. Beshey 2014; post-dates earlier reviews. The LOS was stated as NR but in the original it is 15 days
3. Brun-Buisson 1989; 47 patients excluded because they did not match the inclusion criteria. This is a three-arm study with the third arm (non-concurrent control) not included in the analysis in either review.
4. Camus 2005 is a four-arm study with the third and fourth arm not included in the analysis. The LOS was stated as NR in either review, but LOS recovered from original publication.
5. Cerra 1992; 2 patients excluded because they stayed in the ICU less than 5 days. The LOS was stated as NR in either review, but LOS recovered from original publication.
6. de Smet 2009; post-dates earlier reviews. This is a three-arm cluster randomized trial using non-concurrent controls. LOS recovered from original publication, given as not reported in Minozzi. The sample size of the CRT is adjusted to an effective sample size in Minozzi. The discrepant control group counts are uneplxained.
7. Koeman 2006 is a three-arm study with topical chlorhexidine as the third arm. Mortality data recoverable from another systematic review [Melsen WG, Rovers MM, Groenwold RH, Bergmans DC, Camus C, Bauer TT, Hanisch EW, Klarin B, Koeman M, Krueger WA, Lacherade JC. Attributable mortality of ventilator-associated pneumonia: a meta-analysis of individual patient data from randomised prevention studies. Lancet infect Dis. 2013;13(8):665-71].
8. Korinek 1993; 68 excluded because length of stay shorter than 5 days
9. Pneumatikos 2002; 18 patients were excluded: 7 extubated, 4 developed pneumonia during the first 48 hours of mechanical ventilation, 1 underwent tracheotomy, and 6 died. It is not reported if these events occurred before or after the first 5 days of mechanical ventilation.
10. Pugin 1991; 27 patients excluded due to early extubation or early death.
11. Quinio 1996; The LOS in the original publication is used whereas in both reviews this is 20.5 days. Discrepant denominator count for control group unexplained.
12. Rodriguez-Roldan 1990; The LOS in the original publication is used whereas in both reviews this is given as 13.5 days.
13. Unertl 1987; The LOS in the original publication is used whereas in both reviews this is stated as not reported.
14. Wiener 1995; 60 patients excluded (31 early extubation, 20 early deaths, 4 transfer to general medical ward, 1 protocol violation)
15. Chaari 2014; post-dates the Liberati 2009 systematic review. The LOS was stated as NR, but LOS recovered from original publication.
16. Ferrer 1994; 21 exclusions (14 early extubations, six early deaths & one transfer). The counts in both Liberati and Minozzi differs from that in the original publication where the counts of pneumonia were 7/39 and 10/41 in intervention and control groups, respectively and the mortality counts were 12/39 and 11/41, respectively and the ICU LOS was 15.3 (intervention) and 14.3 (control).
17. Gaussorgues 1991; The LOS was stated as NR, but LOS recovered from original publication.
18. Hammond 1992; 82 patients who were excluded (78 short stay, three protocol violation, one unknown)
19. Laggner 1994; 21 patients who were excluded (18 short MV, three protocol violation)
20. Lingnau 1997; is a three-arm study with the two intervention arms combined in Minozzi 2019. The discrepancy in counts is not explained.
21. Palomar 1997; is a three-arm study with 16 exclusions (seven early extubations, ﬁve early deaths, three protocol violation, one other). The third arm was excluded from both systematic reviews as this arm was the only arm to received sucralfate as a trial intervention together with cefotaxime (as PPAP).
22. Stoutenbeek 1996 (published only as an abstract). There are 32 exclusions (25 early extubations, four early deaths, three other).

**Table S3: Pneumonia, mortality & LOS data for Cochrane reviews of TAP + PPAP**

|  |  |  |  | Liberati 2009 & D’amico 1998 | | | |  |  | Minozzi 2021 | | | |  |  |
| --- | --- | --- | --- | --- | --- | --- | --- | --- | --- | --- | --- | --- | --- | --- | --- |
|  |  |  |  | intervention | | control | | original | original | intervention | | control | | Minozzi 2021 | Minozzi 2021 |
| author | Year | Ref | Notes | n | N | n | N |  |  | n | N | n | N |  |  |
| Pneumonia | |  |  |  |  |  |  |  |  |  |  |  |  |  |  |
| Abele-Horn | 1997 | 43 | a | 13 | 58 | 23 | 30 | 19.3 | 19.3 | 13 | 58 | 23 | 30 | 18 | 22 |
| Aerdts | 1991 | 44 | b | 1 | 28 | 29 | 60 | 30 | 23 | 1 | 18 | 29 | 39 | 16 | 16 |
| Blair | 1991 | 45 | c | 12 | 161 | 38 | 170 | 5 | 5 | 12 | 126 | 38 | 130 | 5 | 5 |
| Boland | 1991 | 46 | d | 14 | 32 | 17 | 32 | 8 | 8 | 3 | 15 | 7 | 15 | 8 | 8 |
| Cockerill | 1992 | 47 | e | 4 | 75 | 12 | 75 | 10 | 12 | 4 | 75 | 12 | 75 | 4.5 | 4.5 |
| de la Cal | 2005 | 49 | g |  |  |  |  |  |  | 18 | 53 | 26 | 54 | 30.6 | 33.6 |
| Finch | 1991 | 50 | i | 4 | 20 | 7 | 24 | NR | NR | 4 | 20 | 7 | 24 | NR | NR |
| Jacobs | 1992 | 51 | j | 0 | 45 | 4 | 46 | 9 | 10 | 0 | 36 | 4 | 43 | 9 | 10 |
| Kerver | 1988 | 52 | k | 5 | 49 | 31 | 47 | 17 | 20 | 5 | 49 | 31 | 47 | NR | NR |
| Krueger | 2002 | 53 | l | 91 | 265 | 149 | 262 | 10 | 10 | 91 | 265 | 149 | 262 | NR | NR |
| Palomar | 1997 | 54 | m | 10 | 50 | 25 | 49 | 8 | 8 | 10 | 41 | 25 | 42 | 10 | 10 |
| Rocha | 1992 | 55 | n | 7 | 47 | 25 | 54 | 19 | 18 | 7 | 47 | 25 | 54 | 8 | 8 |
| Sanchez-Garcia | 1998 | 56 |  | 32 | 131 | 60 | 140 | 13 | 13 | 32 | 131 | 60 | 140 | 13 | 13 |
| Stoutenbeek | 2007 | 57 | o | 62 | 201 | 100 | 200 | 13 | 12 | 62 | 201 | 100 | 200 | 13 | 12 |
| Ulrich | 1989 | 58 | p | 7 | 55 | 26 | 57 | 17 | 13 | 7 | 48 | 26 | 52 | 10 | 10 |
| Verwaest | 1997 | 59 | q | 22 | 193 | 40 | 185 | 19.6 | 17 | 22 | 193 | 40 | 185 | 19.6 | 19.6 |
| Verwaest | 1997 | 59 | q | 31 | 200 | 40 | 185 |  |  |  |  |  |  |  |  |
| Winter | 1992 | 60 | r | 3 | 91 | 17 | 92 | 6.4 | 8 | 3 | 91 | 17 | 92 | 4 | 4 |

Abbreviations; n = numerator count; N = denominator count

**Table S3: Pneumonia, mortality & LOS data for Cochrane reviews of TAP + PPAP (Continued)**

|  |  |  |  | Liberati 2009 & D’amico 1998 | | | |  |  | Minozzi 2021 | | | |  |  |
| --- | --- | --- | --- | --- | --- | --- | --- | --- | --- | --- | --- | --- | --- | --- | --- |
|  |  |  |  | intervention | | control | | original | original | intervention | | control | | Minozzi 2021 | Minozzi 2021 |
| author | Year | Ref | Notes | n | N | n | N | LOS | LOS | n | N | n | N | LOS | LOS |
| Mortality | |  |  |  |  |  |  |  |  |  |  |  |  |  |  |
| Abele-Horn | 1997 | 43 | a | 11 | 58 | 5 | 30 | 19.3 | 19.3 | 11 | 58 | 5 | 30 | 18 | 22 |
| Aerdts | 1991 | 44 | b | 4 | 28 | 12 | 60 | 30 | 23 | 4 | 18 | 4 | 39 | 16 | 16 |
| Blair | 1991 | 45 | c | 24 | 161 | 32 | 170 | 5 | 5 | 17 | 126 | 22 | 130 | 5 | 5 |
| Boland | 1991 | 46 | d | 2 | 32 | 4 | 32 | 8 | 8 | 2 | 15 | 4 | 15 | 8 | 8 |
| Cockerill | 1992 | 47 | e | 11 | 75 | 16 | 75 | 10 | 12 | 11 | 75 | 16 | 75 | 4.5 | 4.5 |
| de Jonge | 2003 | 48 | f | 113 | 466 | 146 | 468 | 6.8 | 8.5 | 113 | 466 | 146 | 468 | 6.8 | 8.5 |
| de la Cal | 2005 | 49 | g |  |  |  |  |  |  | 6 | 53 | 15 | 54 | 30.6 | 33.6 |
| de Smet SDD | 2009 | 61 | h |  |  |  |  |  |  | 258 | 805 | 249 | 783 | 9 | 9 |
| Finch | 1991 | 50 | i | 15 | 24 | 10 | 25 | NR | NR | 15 | 20 | 10 | 24 | NR | NR |
| Jacobs | 1992 | 51 | j | 14 | 45 | 23 | 46 | 9 | 10 | 14 | 36 | 23 | 43 | 9 | 10 |
| Kerver | 1988 | 52 | k | 14 | 49 | 15 | 47 | 17 | 20 | 14 | 49 | 15 | 47 | NR | NR |
| Krueger | 2002 | 53 | l | 52 | 265 | 75 | 262 | 10 | 10 | 52 | 265 | 75 | 262 | NR | NR |
| Palomar | 1997 | 54 | m | 14 | 50 | 14 | 49 | 8 | 8 | 10 | 41 | 13 | 42 | 10 | 10 |
| Rocha | 1992 | 55 | n | 27 | 74 | 40 | 77 | 19 | 18 | 10 | 47 | 24 | 54 | 8 | 8 |
| Sanchez-Garcia | 1998 | 56 |  | 51 | 131 | 65 | 140 | 13 | 13 | 51 | 131 | 65 | 140 | 13 | 13 |
| Stoutenbeek | 2007 | 57 | o | 42 | 201 | 44 | 200 | 13 | 12 | 42 | 201 | 44 | 200 | 13 | 12 |
| Ulrich | 1989 | 58 | p | 22 | 55 | 33 | 57 | 17 | 13 | 15 | 48 | 28 | 52 | 10 | 10 |
| Verwaest | 1997 | 59 | q | 47 | 220 | 40 | 220 | 19.6 | 17 | 34 | 193 | 31 | 185 | 19.6 | 19.6 |
| Verwaest | 1997 | 59 | q | 45 | 220 |  |  |  |  |  |  |  |  |  |  |
| Winter | 1992 | 60 | r | 33 | 91 | 40 | 92 | 6.4 | 8 |  |  |  |  | 4 | 4 |

**Table S3 footnotes;**

1. Abele-Horn, 1997; 19 (15%) patients excluded for protocol violation (12 experimental, 7 control).
2. Aerdts, 1991; 32 patients who were excluded from the published paper (16 early extubation, seven early deaths, ﬁve protocol violation, three ‘other’, one

unknown). Two control groups are aggregated in the analysis

1. Blair, 1991; 75 patients excluded because length of stay was less than 48 hours
2. Boland, 1991; 32 patients excluded because they did not remain intubated for 5 days
3. Cockerill, 1992; 10 patients dropped out of the SDD group because of dislike or AEs. All included in the analysis. The LOS obtained from the original publication.
4. de Jonge; 2003; 102 patients excluded because consent was denied or unable to ask consent. The study has non-concurrent controls.
5. de la Cal, 2005; 10 patients excluded because length of stay was less than 3 days
6. de Smet, 2009; Dropout due to non-compliance: SOD: 4.3%, SDD 2.5%. All patients included in the analysis. This is a three-arm study with the third arm not included in the analysis. LOS recovered from original publication, given as not reported in Minozzi. The sample size of the CRT is adjusted to an effective sample size in Minozzi. The discrepant control group counts are uneplxained.
7. Finch, 1991; A total of 49 patients were admitted, of whom 44 (90%) were evaluated; reasons for exclusion not provided.
8. Jacobs, 1992; 11 patients excluded because length of stay shorter than 3 days, 1 suspected to be an HIV carrier.
9. Kerver; LOS data recovered from the original publication as was given as not reported in both systematic reviews.
10. Krueger, 2002; 19 (3.4%) patients were excluded after enrolment
11. Palomar, 1997; 22 patients excluded because early extubation (10), early death (8), protocol violation (3), cefotaxime-related hypertensive reaction (1). This is a three-arm study with the third arm not included in the analysis.
12. Rocha, 1992; 50 excluded (15 early extubations, 31 early deaths, 2 protocol violation, 2 other). LOS data recovered from the original publication. Both reviews both give the mean LOS as 8 days.
13. Stoutenbeek, 2007; 4 patients were excluded from the final analysis after randomisation
14. Ulrich, 1989; 12 patients excluded because they died within 24 hours. LOS data recovered from the original publication as this was given as 10 days in both systematic reviews.
15. Verwaest, 1997; In 40 patients (6%; balanced between groups), a correct evaluation was impossible because important clinical or bacteriological data were missing. This is a three-arm study with the third arm not included in the analysis. Additional data was recovered from the Verhagen MD thesis.
16. Winter 1992 is a three-arm study with the third arm not included in the analysis. The mortality data for this study is missing from Minozzi. LOS data recovered from the original publication as this was given as 4 days in both systematic reviews.

**References**

1. Bellissimo‐Rodrigues F, Bellissimo‐Rodrigues WT, Viana JM, Teixeira GC, Nicolini E, Auxiliadora‐Martins M, et al. Effectiveness of oral rinse with chlorhexidine in preventing nosocomial respiratory tract infections among intensive care unit patients. Infect Cont Hosp Epidemiol2009;30(10):952‐8.
2. Berry AM, Davidson PM, Masters J, Rolls K, Ollerton R. Effects of three approaches to standardized oral hygiene to reduce bacterial colonization and ventilator associated pneumonia in mechanically ventilated patients: A randomised control trial. Internat J Nursing Studies 2011;48(6):681–8.
3. Cabov T, Macan D, Husedzinovic I, Skrlin‐Subic J, Bosnjak D, Sestan‐Crnek S, et al. The impact of oral health and 0.2% chlorhexidine oral gel on the prevalence of nosocomial infections in surgical intensive‐care patients: a randomized placebo‐controlled study. Wiener Klinische Wochenschrift 2010;122(13‐14):397‐404.
4. Chen QL, Ye XF, Jiang YZ, Yan MQ. Application of new oral care method to orotracheal intubation. *Fujian Med J* 2008;**30**(5):155–7.
5. DeRiso AJ, Ladowski JS, Dillon TA, Justice JW, Peterson AC. Chlorhexidine gluconate 0.12% oral rinse reduces the incidence of total nosocomial respiratory infection and nonprophylactic systemic antibiotic use in patients undergoing heart surgery. Chest 1996;109(6):1556‐61.
6. Fourrier F, Cau‐Pottier E, Boutigny H, Roussel‐Delvallez M, Jourdain M, Chopin C. Effects of dental plaque antiseptic decontamination on bacterial colonization and nosocomial infections in critically ill patients. Inten Care Med 2000;26:1239‐47.
7. Fourrier F, Dubois D, Pronnier P, Herbecq P, Leroy O, Desmettre T, et al. Effect of gingival and dental plaque antiseptic decontamination on nosocomial infections acquired in the intensive care unit: a double‐blind placebo‐controlled multicenter study. Crit Care Med 2005;33(8):1728‐35.
8. Fu T, Zhong Q, Zheng C. Bacteriostasis effect of oral administration of chlorhexidine on patients with mechanical ventilation and prevention and treatment of ventilator - associated pneumonia. Chinese Nursing Research 2019;33(3):431-4.
9. Grap MJ, Munro CL, Hamilton VA, Elswick RK Jr, Sessler CN, Ward KR. Early, single chlorhexidine application reduces ventilator-associated pneumonia in trauma patients. *Heart & Lung* 2011;**40**(5):e115–22.
10. Jacomo AD, Carmona F, Matsuno AK, Manso PH, Carlotti AP. Effect of oral hygiene with 0.12% chlorhexidine gluconate on the incidence of nosocomial pneumonia in children undergoing cardiac surgery. Infect Cont Hosp Epidemiol 2011;32(6):591‐6.
11. Koeman M, Van der Ven AJ, Hak E, Joore HC, Kaasjager K, De Smet AG, et al. Oral decontamination with chlorhexidine reduces the incidence of ventilator-associated pneumonia. Amer J Resp Crit Care Med 2006;173(12):1348-55.
12. Kusahara DM, Peterlini MA, Pedreira ML. Oral care with 0.12% chlorhexidine for the prevention of ventilator‐associated pneumonia in critically ill children: Randomised, controlled and double blind trial. International J Nursing Studies 2012;49(11):1354‐63.
13. Meidani M, Khorvash F, Abbasi S, Cheshmavar M, Tavakoli H. Oropharyngeal irrigation to prevent ventilator-associated pneumonia: comparing potassium permangenate with chlorhexidine. Internat J Preventive Med 2018;**9**(1):93.
14. Meinberg MC, Cheade M de F, Miranda AL, Fachini MM, Lobo SM. The use of 2% chlorhexidine gel and toothbrushing for oral hygiene of patients receiving mechanical ventilation: effects on ventilator‐associated pneumonia [Uso de clorexidina 2% gel e escovacao mecanica na higiene bucal de pacientes sob ventilacao mecanica: efeitos na pneumonia associada a ventilador]. Revista Brasileira de Terapia Intensiva 2012;24(4):369‐74
15. Munro CL, Grap MJ, Jones DJ, McClish DK, Sessler CN. Chlorhexidine, toothbrushing, and preventing ventilator‐associated pneumonia in critically ill adults. American J Crit Care 2009;18(5):428‐37.
16. Ozcaka O, Basoglu OK, Buduneli N, Tasbakan MS, Bacakoglu F, Kinane DF. Chlorhexidine decreases the risk of ventilator‐associated pneumonia in intensive care unit patients: a randomized clinical trial. J Periodontal Res 2012;47(5):584‐92.
17. Scannapieco FA, Yu J, Raghavendran K, Vacanti A, Owens SI, Wood K, et al. A randomized trial of chlorhexidine gluconate on oral bacterial pathogens in mechanically ventilated patients. Crit Care 2009;13(4):R117.
18. Sebastian MR, Lodha R, Kapil A, Kabra SK. Oral mucosal decontamination with chlorhexidine for the prevention of ventilator‐associated pneumonia in children ‐ a randomized, controlled trial. Pediatric Crit Care Med 2012;13(5):e305‐10.
19. Tantipong H, Morkchareonpong C, Jaiyindee S, Thamlikitkul V. Randomized controlled trial and meta‐analysis of oral decontamination with 2% chlorhexidine solution for the prevention of ventilator‐associated pneumonia. Infect Cont Hosp Epidemiol 2008;29(2):131‐6.
20. Tuon FF, Gavrilko O, Almeida S, Sumi ER, Alberto T, Rocha JL, et al. Prospective, randomised, controlled study evaluating early modification of oral microbiota following admission to the intensive care unit and oral hygiene with chlorhexidine. *J Global Antimicrob Resist* 2017;**8**:159-63.
21. Bergmans DCJJ, Bonten MJM, Gailard CA, Paling JC, van der Geest S, van Tiel FH, et al. Prevention of ventilator‐associated pneumonia by oral decontamination: a prospective randomized, double blind, placebo controlled study. Am J Respir Crit Care Med 2001;164(3):382‐8.
22. Beshey BN, Okasha AS, Eldin ME. Fluconazole and selective digestive decontamination for prevention of Candida infection in high risk critically ill patients. Alexandria J Med. 2014 ;50(1):93-8.
23. Brun‐Buisson C, Legrand P, Rauss A, Richard C, Montravers F, Besbes M, et al. Intestinal decontamination for control of nosocomial multiresistant Gram‐negative bacilli. Ann Intern Med 1989;110:873‐81.
24. Camus C, Bellissant E, Sebille W, Perrotin D, Garo B, Legras A, et al. Prevention of acquired infections in intubated patients with combination of two decontaminations regimens. Crit Care Med 2005;33(2):307‐14.
25. Cerra FB, Maddaus MA, Dunn DL, Wells CL, Konstantinides NN, Lehmann SL, Mann HJ. Selective gut decontamination reduces nosocomial infections and length of stay but not mortality or organ failure in surgical intensive care unit patients. Arch Surgery. 1992 ;127(2):163-9.
26. Gastinne H, Wolff M, Delatour F, Faurisson F, Chevret S. A controlled trial in intensive care units of selective decontamination of the digestive tract with nonabsorbable antibiotics. N Engl J Med 1992;326:594‐9.
27. Georges B, Mazerolles M, Decun JF, Rouge P, Pomies S, Cougot P, et al. Décontamination digestive sélective résultats d'une ètude chez le polytraumatisé. Réan Urg 1994;3:621‐627.
28. Koeman M, van der Ven AJ, Hak E, et al. Oral decontamination with chlorhexidine reduces the incidence of ventilator-associated pneumonia. *Am J Respir Crit Care Med* 2006;173:1348-1355
29. Korinek AM, Laisne MJ, Raskine L, Deroin V, Sanson‐Lepors MJ. Selective decontamination of the digestive tract in neurosurgical care units patients: a double blind, randomized, placebo‐controlled study. Crit Care Med 1993;21:1466‐73.
30. Pneumatikos I, Koulouras V, Nathanail C, Goe D, Nakos G. Selective decontamination of subgloottic area in mechanically ventilated patients with multiple trauma. Inten Care Med 2002;28:432‐7.
31. Pugin J, Auckenthaler R, Lew DP, Suter PM. Oropharyngeal decontamination decreases incidence of ventilator‐associated pneumonia. JAMA 1991;265:2704‐10.
32. Quinio B, Albanèse J, Bues‐Charbit M, Viviand X, Martin C. Selective Decontamination of the digestive tract in multiple trauma patients: prospective, double blind, randomised, placebo‐controlled study. Chest 1996;109:765‐72.
33. Rodrìguez‐Roldàn JM, Altuna‐Cuesta A, Lòpez A, Carrillo A, Garcia J, Leòn J, Martìnez‐Pellùs AJ. Prevention of nosocomial lung infection in ventilated patients:use of an antimicrobial pharyngeal nonabsorbable paste. Crit Care Med 1990;18:1239‐42.
34. Unertl K, Ruckdeschel G, Selbmann HK, Jensen U, Forst H, Lenhart FP, et al. Prevention of colonization and respiratory infections in long term ventilated patients by local antimicrobial prophylaxis. Intensive Care Med 1987;13:106‐13.
35. Wiener J, Itokazu G, Nothan C, Kabins SA, Weinstein RA. A randomized, double‐blind, placebo‐controlled trial of selective digestive decontamination in a medical‐surgical intensive care unit. Clin Infect Dis 1995;20:861‐7.
36. Chaari A, Zribi E, Dammak H, Ghadoun H, Chtara K, Sfar S, et al. Does selective digestive decontamination prevent ventilatorassociated pneumonia in trauma patients? American Journal of Therapeutics 2014;21:470-6.
37. Ferrer M, Torres A, Gonzàles J, de la Bellacasa JP, El‐Ebiary M, Roca M, et al. Utility of selective digestive decontamination in mechanically ventilated patients. Ann Intern Med 1994;120:389‐95.
38. Gaussorgues P, Salord F, Sirodot M, Tigaud S, Cagnin S, Gerard M, et al. Efficacité de la décontamination digestive sur la survenue des bactériémies nosocomiales chez les patients sous ventilation mécanique et recevant des betamimétiques. Réan Soins Intens Méd Urg 1991;7:169‐74.
39. Hammond JMJ, Potgieter PD, Saunders GL, Forder AA. Double blind study of selective decontamination of the digestive tract in intensive care. Lancet 1992;340:5‐9.
40. Laggner AN, Tryba M, Georgopoulos A, Lenz K, Grimm G, Graninger W, et al. Oropharyngeal decontamination with gentamicin for long‐term ventilated patients on stress ulcer prophylaxis with sucralfate?. Wien Klin Wochenschr 1994;106:15‐19.
41. Lingnau W, Berger J, Javorsky F, Lejeune P, Mutz N, Benzer H. Selective intestinal decontamination in multiple trauma patients: prospective, controlled trials. J Trauma 1997;42:687‐694.
42. Stoutenbeek CP, Van Saene HKF, Zandstra DF. Prevention of multiple organ failure by selective decontamination of the digestive tract in multiple trauma patients. In: Immune consequences of trauma, shock and sepsis. Mechanisms and therapeutic approaches eds; Faist E, Baue AE, Schildberg FW. Pabst Science Publishers, Berlin, 1996;2:1055‐66.
43. Abele‐Horn M, Dauber A, Bauernfeind A, Russwurm W, Seyfarth‐Metzger I, Gleich P, Ruckdeschel G. Decrease in nosocomial pneumonia in ventilated patients by selective oropharyngeal decontamination (SOD). Intensive Care Med 1997;23:187‐195.
44. Aerdts SJA, van Dalen R, Clasener HAL, Festen J, van Lier HJJ, Vollaard EJ. Antibiotic prophylaxis of respiratory tract infection in mechanically ventilated patients. Chest 1991;100:783‐91.
45. Blair P, Rowlands BJ, Lowry K, Webb H, Amstrong P, Smilie J. Selective decontamination of the digestive tract: a stratified, randomized, prospective study in a mixed intensive care unit. Surgery 1991;110:303‐10.
46. Boland JP, Sadler DL, Stewart W, Wood DJ, Zerick W, Snodgrass KR. Reduction of nosocomial respiratory tract infections in the multiple trauma patients requiring mechanical ventilation by selective parenteral and enteral antisepsis regimen (SPEAR) in the intensive care. XVII Congress of Chemotherapy,1991.
47. Cockerill FR, Muller SR, Anhalt JP, Marsh HM, Farnell MB, Mucha P, et al. Prevention of infection in critically ill patients by selective decontamination of the digestive tract. Ann Intern Med 1992;117:545‐53
48. de Jonge E, Schultz MJ, Spanjaard L, Bossuyt PM, Vroom MB, Dankert J, Kesecioglu J. Effects of selective decontamination of digestive tract on mortality and acquisition of resistant bacteria in intensive care: a randomised controlled trial. The Lancet. 2003; 362(9389):1011-6.
49. de_la Cal_MA, Cerdà_E, Garcia-Hierro_P, van_Saene_HK, Gómez- Santos_D, Negro_E, et al. Survival benefit in critically hill burned patients receiving selective decontamination of the digestive tract. A randomized, placebo-controlled, double-blind trial. *Annals of Intern Med* 1992;**117**:545-53.
50. Finch RG, Tomlinson P, Holliday M, Sole K, Stack C, Rocker G. Selective decontamination of the digestive tract (SDD) in the prevention of secondary sepsis in a medical/surgical intensive care unit. XVII International Congress Chemotherapy,1991.
51. Jacobs S, Foweraker JE, Roberts SE. Effectiveness of selective decontamination of the digestive tract (SDD) in an ICU with a policy encouraging a low gastric pH. Clin Intens Med 1992;3:52‐8.
52. Kerver AJH, Rommes JH, Mevissen‐Verhage EAE, Hulstaert PF, Vos A, Verhoef J, et al. Prevention of colonization and infection in critically ill patients: A prospective randomized study. Crit Care Med 1988;16:1087.
53. Krueger WA, Lenhart FP, Neeser G, Ruckdeschel G, Schreckhase H, Eissner HJ, et al. Influence of combined intravenous and topical antibiotic prophylaxis on the incidence of infections, organ dysfunctions and mortality in critically ill surgical patients. Am J Respir Crit Care Med 2002;166:1029‐37.
54. Palomar M, Alvarez‐Lerma F, Jorda R, Bermejo B. Prevention of nosocomial infection in mechanically ventilated patients: Selective digestive decontamination versus sucralfate. Clin Intens Care 1997;8:228‐35.
55. Rocha LA, Martin MJ, Pita S, Paz J, Seco C, Margusino L, et al. Prevention of nosocomial infection in critically ill patients by selective decontamination of digestive tract. Inten Care Med 1992;18:398‐404.
56. Sanchez‐Garcia M, Cambronero JA, Lopez J, Cerda E, Rubio J, et al. Effectiveness and cost of selective decontamination of the digestive tract (SDD) in critically ill intubated patients. A randomized, double blind, placebo‐controlled, multicentric trial.. Am J Respir Crit Care Med 1998;158:908‐16.
57. Stoutenbeek CP, Van Saene HKF, Little RA, Whitehead A. The effect of selective decontamination on the digestive tract on mortality in multiple trauma patients: a multicentre randomized controlled trial. Inten Care Med 2007;33:261‐70.
58. Ulrich C, Harinck‐deWeerd JE, Bakker NC, Jacz K, Doornbos L, et al. Selective decontamination of the digestive tract with norfloxacin in the prevention of ICU‐acquired infections: a prospective randomized study. Intensive Care Med 1989;15:424‐31.
59. Verwaest C, Verhaegen J, Ferdinande P, Schets M, Van der Berghe G, Verbist L, et al. Randomized, controlled trial of selective digestive decontamination in 600 mechanically ventilated patients in a multidisciplinary intensive care unit. Crit Care Med 1997;25:63‐71.
60. Winter R, Humphreys H, Pick A, MacGowan AP, Willatts SM, Speller DCE. A controlled trials of selective decontamination of the digestive tract in intensive care and its effect on nosocomial infection. J Antimicrob Chemother 1992;30:73‐87.
61. de Smet AM, Kluytmans JA, Cooper BS, Mascini EM, Benus RF, Van der Werf TS, Van der Hoeven JG, Pickkers P, Bogaers-Hofman D, Van Der Meer NJ, Bernards AT. Decontamination of the digestive tract and oropharynx in ICU patients. N Engl J Med. 2009 ;360(1):20-31.
62. Panchabhai TS, Dangayach NS, Krishnan A, Kothari VM, Karnad DR. Oropharyngeal cleansing with 0.2% chlorhexidine for prevention of nosocomial pneumonia in critically ill patients: an open‐label randomized trial with 0.01% potassium permanganate as control. Chest 2009;135(5):1150‐6.
